# An Open Software Platform for the Automated Design of Paper-Based Microfluidic Devices

**DOI:** 10.1038/s41598-017-16542-8

**Published:** 2017-11-24

**Authors:** Nicholas S. DeChiara, Daniel J. Wilson, Charles R. Mace

**Affiliations:** Department of Chemistry, Tufts University, 62 Talbot Avenue, Medford, MA 02155 USA

## Abstract

Paper-based microfluidic devices have many applications in biomedical and environmental analysis. However, the process of prototyping device designs can be tedious, error-prone, and time-consuming. Here, we present a cross-platform, open-source software tool—AutoPAD—developed to quickly create and modify device designs and provide a free alternative to commercial design software. The capabilities that we designed to be inherent to AutoPAD (e.g., automatic zone alignment and design refactoring) highlight its potential use in nearly any paper-based microfluidic device application and for creating nearly any desired design, which we demonstrate through the recreation of numerous device designs from the literature.

## Introduction

In the decade since they were first introduced^1^, paper-based microfluidic devices (also called microfluidic paper-based analytical devices; µPAD) have emerged as an analytical platform that is capable of enabling the development of a wide range of analytical and bioanalytical tests^2–5^. In particular, paper-based microfluidic devices have the advantage of low cost and portability, which make them attractive for creating point-of-care diagnostic assays^6^. As new applications for paper-based microfluidic devices are developed, new device architectures often accompany them. While many assays can be performed using devices composed of a single layer of patterned paper^7–12^, there are numerous advantages to creating three-dimensional paper-based microfluidic devices. Three-dimensional devices, prepared from strategies that employ multiple layers of paper^13^ or a single folded layer of paper^14^, facilitate spatial separation of stored reagents (a requirement for multiplexed assays)^15,16^, performing sequential multistep reactions (e.g., immunoassays, nucleic acid detection)^17–20^, and the incorporation of sophisticated readouts into devices (e.g., barcodes or timers)^21^.

Three-dimensional devices fabricated from multiple sheets of paper, each of which comprise multiple copies of a device layer, rely on procedures that are similar to additive manufacturing processes^22^. The hydrophilic regions that connect layers of paper (e.g., channels and test zones) must overlap exactly to ensure reproducible capillary flow across all devices. Slight errors in device design may propagate significant inconsistencies that ultimately affect the performance of assays. Misalignment of zones in the fluidic pathway of a device, which can result from manufacturing error^22^ or patterning error, can lead to device failure. Consequently, the process of creating, optimizing, and improving upon designs for paper-based microfluidic devices can be slow and tedious. Even a single change to a design may require a complete readjustment of the entire device layout by the user. Devices become substantially more error-prone and time-consuming to fabricate when additional layers or complex channel structures are required. These problems are exacerbated further by the need to produce and test many iterations of the device, which is often required for the development and optimization of an assay. Ultimately, these concerns may limit the evaluation or use of such designs. Diminishing the impact of these factors on prototyping and manufacturing processes would reduce the amount of time and costs invested in creating, modifying, and fabricating devices and provide improved quality assurance for paper-based microfluidic devices.

We recognized that many of these challenges are associated with adapting existing programs that were initially intended for other purposes (e.g., graphic design or slide presentation software). For example, Adobe Illustrator is a popular option for graphic design, but can cost US$20 per month per license-holder^23^. CorelDRAW, another popular design software, costs nearly US$500 per permanent license^24^, while licenses for more powerful and precise drawing platforms can be even more cost prohibitive (e.g., US$695 for SketchUp Pro or US$185 per month per license-holder for AutoCAD)^25,26^. Consequently, we sought to develop a software program that was created purposefully to design and rapidly prototype paper-based microfluidic devices. We named this program AutoPAD—*auto*mated design of *p*aper *a*nalytical *d*evices. In order to ensure accessibility for a broad and global user base, we developed AutoPAD to be a cross-platform, open-source resource. Similar to other open-source scientific software (e.g., ImageJ^27^ and Jmol^28)^, we make the source code for AutoPAD free to use and modify. This approach supports our goals to (i) reduce costs associated with starting and maintaining research laboratories in the field of paper-based diagnostics and (ii) enable new opportunities for researchers for whom costs of other design software may be prohibitive (e.g., high school students and laboratories in developing economies). Further, if device designs are commonly accessible and sharable, which is possible using AutoPAD text file outputs, a significant hurdle to information sharing and collaboration between international laboratories may be overcome^29^. Although AutoPAD does not have as many features and tools as commercial software options, all of its components were designed with developers of paper-based microfluidics in mind. AutoPAD’s streamlined features make it easy to use, and ensuring that the software remains open-source will allow for collaborative enhancement of the program’s capabilities by those who work within the field.

To develop AutoPAD, we adopted a relativistic design methodology whereby a device design is reduced to an interconnected network of objects, which do not have absolute positions but are instead located according to their inter-network connections. Through this organization, changing one object within the design causes all other objects to readjust their positions automatically, which is in stark contrast to conventional methods where each object within a design would need to be readjusted manually by the user. As a result, devices designed using AutoPAD scale well, as the amount of time required to make an adjustment does not change significantly between designs of varying size and complexity. In this manuscript, we demonstrate the development and use of AutoPAD with a number of case studies, each of which were chosen to highlight different capabilities of the software, and which, taken as a whole, show that our system has the capacity to produce almost any desired design.

## Experimental Design

### Design of Software Interface

**Figure 1:**
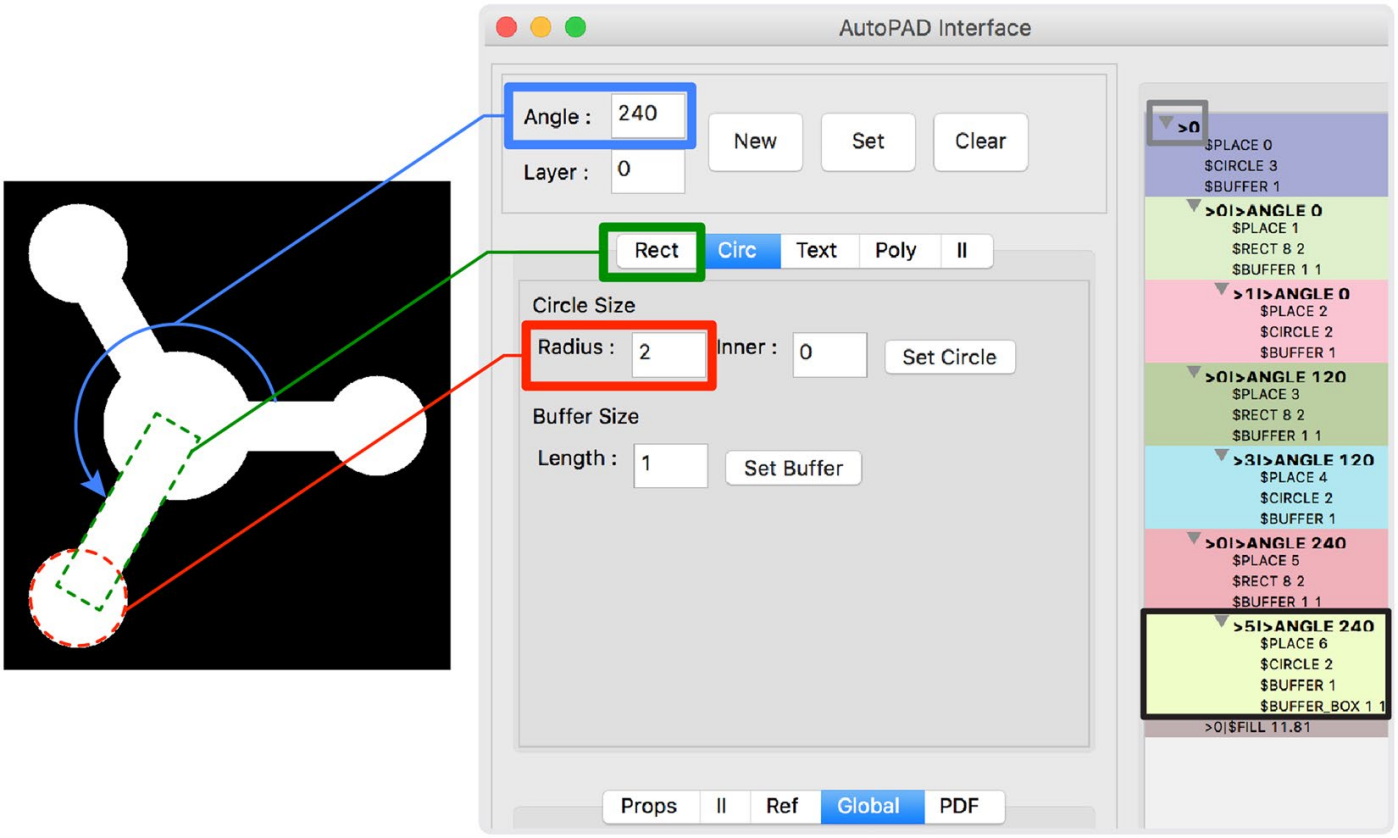
Screenshot of the AutoPAD Tree Interface (right) with a single layer paper-based microfluidic device design shown in preview mode (left). A black box in the Node Tree highlights the commands used to result in the accurate placement of a rectangular channel (green) and circular test zone (red) at a defined angle of 240° (blue) relative to the first node of the device, as denoted by the >0 node command (gray).

**Figure 2:**
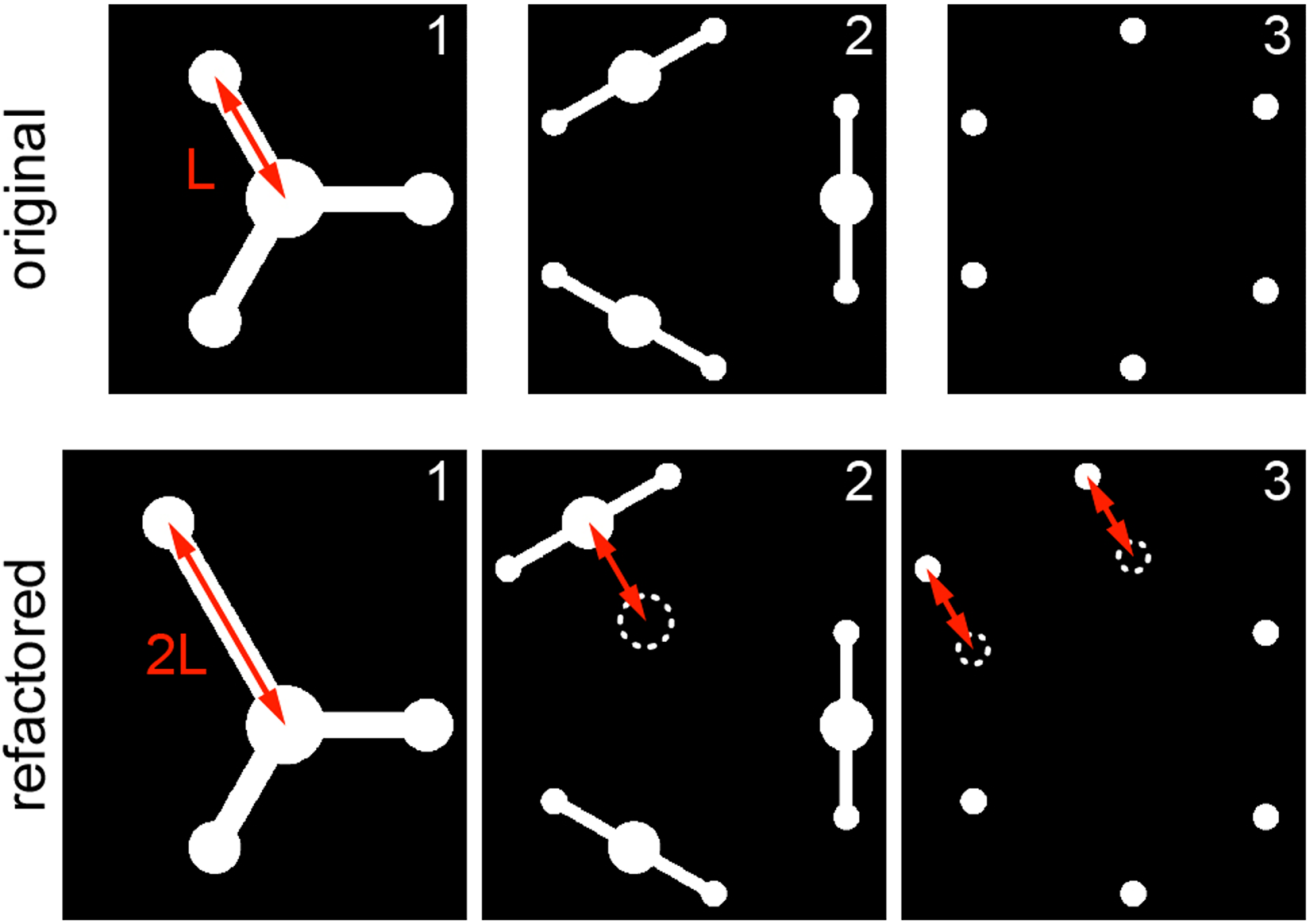
Refactoring of design features occurs automatically across multiple layers of a three-dimensional paper-based microfluidic device. The original device design (top) comprises a first layer (layer 1) patterned with a central sample application zone and three circular zones positioned at a distance, L, using three rectangular channels. Each zone is connected to splitting channel (on layer 2) and finally two test zones (on layer 3), which creates a total of six outputs. If one circular zone on the first layer is moved to a distance 2 L, all connected objects—the linking rectangular channel and geometric shapes positioned on subsequent nodes in layers 2 and 3—are moved the correct distance automatically by AutoPAD.

The basis of the AutoPAD software is script files representing designs. A device could be created simply by writing a script into a text file and passing it through our program. However, we wanted to create a more user-friendly and visual interface that did not require the user to master the underlying scripting language to design devices. In AutoPAD, devices are represented as trees of interconnected shapes, where each node on the tree has child branches and its own parent branch from which it is sourced (Fig. 1). The same structure is used in computer file systems, where files are stored in folders that can be then nested inside of other folders. Recognizing this, we designed a “Tree Interface”, similar to a file browser, where each node is a folder containing its properties, such as a description of its shape and size, as well as all of the nodes attached to it. In this manner, when the properties of one node are modified (e.g., spatial location), then the corresponding properties of all attached nodes will be automatically reconfigured (Fig. 2). Additionally, we constructed the interface to automatically provide previews of the device while it is under construction, and the user may select parts of the device simply by selecting those parts on the preview image. The interface is a self-contained design solution, as it can be used to create, save, load, and compile script files.

### Identification of Desired Features

In order to identify the desired features of AutoPAD, we surveyed the literature for paper-based microfluidic devices and identified commonalities among their designs. Generally, paper-based microfluidic devices consist of paper channels that are either cut out (e.g., two-dimensional paper networks; 2DPN)^30–32^ or patterned with hydrophobic barriers (e.g., using photoresist or wax)^33–35^. We determined that a vast selection of differing designs could be generalized as a series of connected channels, circular zones, and rectangular pathways. As a result, we designed a system that describes devices as lists of these connected shapes, and drew inspiration from the literature to determine which geometric shapes should be supported most prominently with added support for generalized polygons for increased design flexibility. We also identified that text was commonly used in device designs. As such, we added support for rendering text in any size, font style, or rotation. We then looked at the various means of constructing devices, especially multi-layered devices; some device designs are cut out of paper or membranes^32^ or masked using stencils^36^, while many others are constructed using layers of patterned paper and adhesive^37–39^. To address all cases, we developed a system that would automatically generate cutting pattern outlines of device designs, which could be vectorized to work with any cutting plotter robot or laser cutter (Supplementary Information). In order to provide support for wax printing methods, we developed a buffering system whereby a device could be rendered either as white channels upon a black background (i.e., no wax deposited upon printing) or as white channels surrounded by shape-fitting black borders, which use less wax when printing. We also built tools for the construction of origami devices^16,40^ by developing features that allow layers to be arranged into grids, which could then be printed and folded. We recognized that many origami devices consist of the same layer designs rotated and reflected in various arrangements, so we additionally allowed users to reuse existing layers with different transformations rather than needing to redesign a layer several times for each rotation.

Device designs have specific desired dimensions, as it is important to control factors related to experimental performance (e.g., total channel volume), economy (e.g., minimizing material waste), and operability (e.g., holding or manipulating a device). However, an image by itself carries no dimensional data, because it can be printed at varying pixels-per-inch resolutions. Therefore, AutoPAD will compile images into PDF files of set dimensions based on the user’s specifications. Depending on the desired page size and the dimensions of each device, intact layer designs will be tiled across the page as many times as space allows. When these sheets are printed, the device will have the desired dimensions and will be ready for mass assembly. Files of PDF format are recognized by every operating system, every printer, and most graphics design software, which ensures the compatibility of our program and its outputs with other systems currently in use.

## Results and Discussion

### Tutorials and User Documentation

We have prepared extensive documentation in support of training new users of AutoPAD. We provide the following four documents in the Supplementary Information that accompanies this manuscript: (i) *Materials and Methods*. We describe technical details related to programming AutoPAD. Additionally, we include images of case studies for using AutoPAD to recreate several literature examples of paper-based microfluidic devices. (ii) *Glossary*. We provide a list of commands and a description of their uses. (iii) *Introduction to the Tree Interface*. This document instructs the user how to navigate the front-end interface of AutoPAD and highlights important components, panels, and principles required to create a design. (iv) *Getting Started with AutoPAD*. This document guides users through the process of designing paper-based microfluidic devices using AutoPAD—from conceptualization, through the use of commands, and ultimately to generating printable files. In essence, all previous documents support this guide. In addition, we have prepared five narrated tutorial videos in order to help guide new users through learning key functions of AutoPAD and begin to design their own paper-based microfluidic devices. These videos are also available online on the Mace Lab YouTube channel^41^.

### Case Studies

In order to demonstrate the broad and enabling capabilities of AutoPAD, we chose several case studies that highlight functions that are necessary to design, manufacture, and ultimately use paper-based microfluidic devices. Each case study is accompanied by: (i) a figure depicting the literature reference rendered as an image provided by the AutoPAD Interpreter, images of modified paper-based microfluidic devices designed in AutoPAD, and images of devices manufactured using designs created in AutoPAD; and (ii) examples of code lines that may be easily modified to make complex adjustments to design geometry. In these case studies, the appearance (e.g., feature size, border sharpness, or color) of paper-based devices made from designs produced in AutoPAD may differ slightly from their parent software designs. These differences can be attributed to the way that melted wax permeates chromatography paper^34^. We also provide text files (and accompanying PNG and PDF outputs) for all six case studies in a compressed zip file in a GitHub repository^42^, which can serve as the basis for the design of various types of paper-based microfluidic devices.

### Case Study 1. Single Layer Sample Splitting

**Figure 3:**
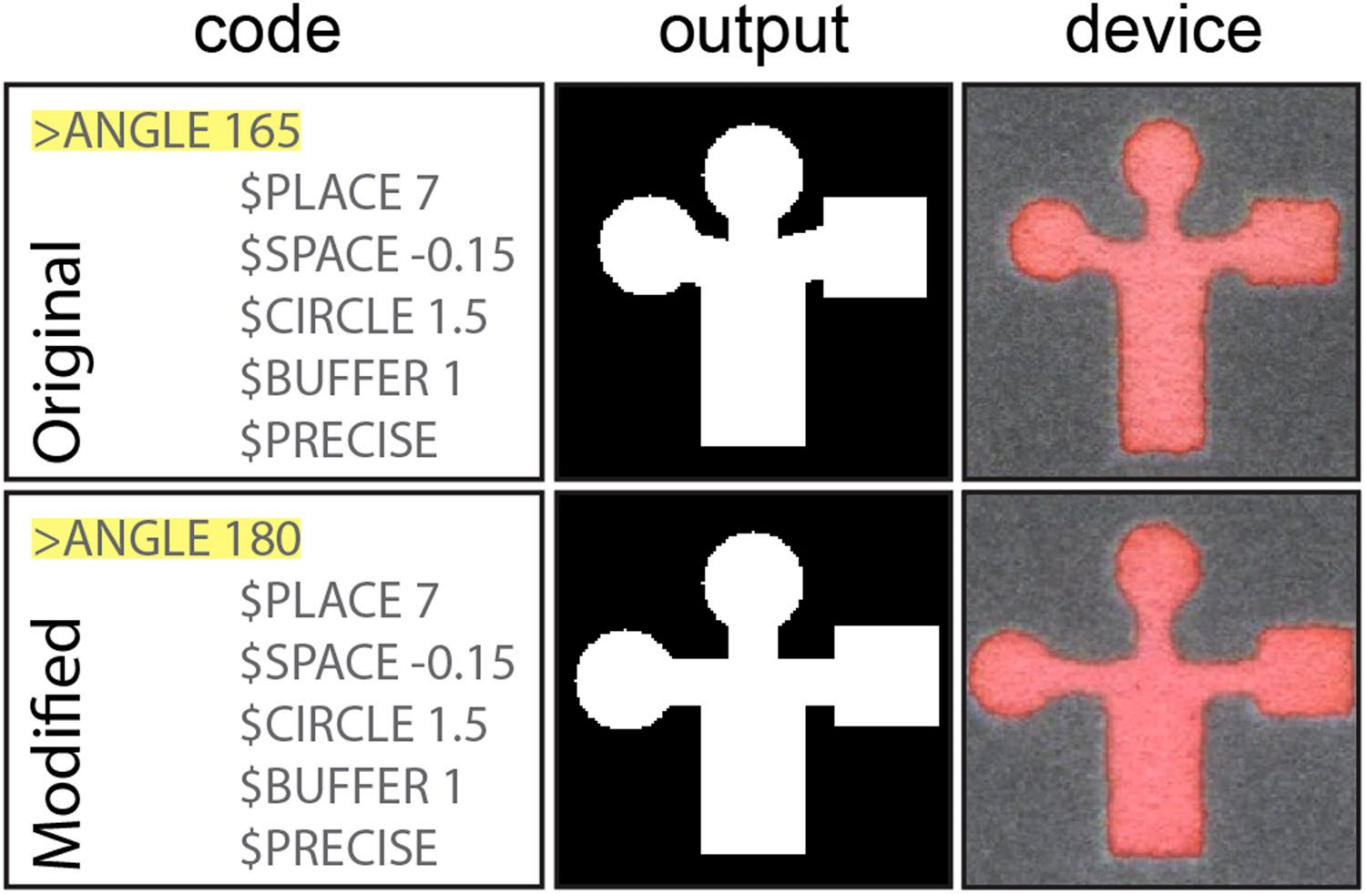
Use of AutoPAD to design a single layer device that splits a sample into three test zones. The left column displays a section of code used to create the design, where the command to be modified is highlighted in yellow. The middle column displays the AutoPAD output (as a PNG file) of the full device design. The right column is an image of a paper-based device fabricated from the AutoPAD design with red dye added into the channels. The top and bottom rows illustrate original and modified designs. Adapted from Martinez *et al*.^1^.

The case of splitting one sample into three test zones on a single layer of paper represents the design used in the first report of patterned paper for bioassays by Whitesides and coworkers^1^. We highlight this design here because it also demonstrates many of the basic features of AutoPAD, such as the placement of geometric shapes and angles (Fig. 3, Case Study 1). Additionally, this design showcases the “snap-to-shape” buffer system that allows for clean outlining of continuous hydrophilic channels prepared from multiple discrete objects. Modifications to the design of these devices (e.g., the length of channels or shape of test zones) require changing only two or three numbers using the Tree Interface or within the script (Fig. 3). AutoPAD will automatically adjust the positions of relevant objects and generate new image files with minimal user input, which greatly facilitates the manufacture and testing of paper-based microfluidic devices.

### Case Study 2. Paper Microzone Plates

Patterned paper microzone plates were developed as low-cost alternatives to standard 96- and 384-well plastic microwell plates^43^. Because these microzone plates can be printed on-demand and offer users the flexibility to make design modifications (e.g., zone geometry or introduction of channels), numerous applications including paper-based ELISA^44–46^, array-based screening^47^, and development of tissue engineering scaffolds^48,49^ are made possible. We recreated a 96-well paper microzone plate in order to demonstrate how AutoPAD can be used to create arrays of geometric shapes and incorporate text labels (Supplementary Fig. S1, Case Study 2). This latter capability is additionally useful for providing users with clear identifiers for test zones of multiplexed assays (e.g., for unique markers or to differentiate tests from controls)^50,51^.

### Case Study 3. Two-Dimensional Paper Networks (2DPN)

Two-dimensional paper networks (2DPN) demonstrate both a basic application of layers and also the use of cut layers (Supplementary Fig. S2, Case Study 3)^52^. Unlike the previous case studies, this design requires the porous media (e.g., nitrocellulose membranes) to be laser cut instead of patterned with hydrophobic barriers. The AutoPAD interpreter is able to generate outlines using the $CUT property, which creates linewidths that can be traced into paths that are compatible with laser cutter software. Designs generated using the $CUT property can also be turned into paths compatible with robotic knife plotters, and used to cut layers of double-sided adhesive that facilitate the manufacture of three-dimensional paper-based microfluidic devices^22^.

### Case Study 4. Three-Dimensional Paper-Based Immunoassays

We have recently developed a general device architecture that enables the manufacture of three-dimensional paper-based immunoassays in both singleplex^17,53^ and multiplex^54^ formats. Each of these devices comprises multiple layers of paper and double-sided adhesive. For example, our sandwich immunoassay device for human chorionic gonadotropin (i.e., pregnancy hormone) is prepared from five layers of patterned paper or nylon membrane and an additional five layers of patterned adhesive film to affix these layers together^17^. We chose to recreate this device using AutoPAD to showcase the capability of inherent alignment of zones between multiple discrete layers (Supplementary Fig. S3, Case Study 4). Additionally, the AutoPAD interpreter automatically generates cut layers required to patterned adhesive films. Unlike the original immunoassay device design, which we created using Adobe Illustrator, AutoPAD can be used to produce design modifications rapidly (e.g., altering the length of the lateral channel) by changing only two or fewer numbers in the Interpreter user interface. AutoPAD will then automatically reposition all connected nodes on all layers to match the adjustment, scale the total image size, align the layers, rebuild cut layers, and generate printable image files of all layers.

### Case Study 5. Three-Dimensional Sample Splitting

**Figure 4:**
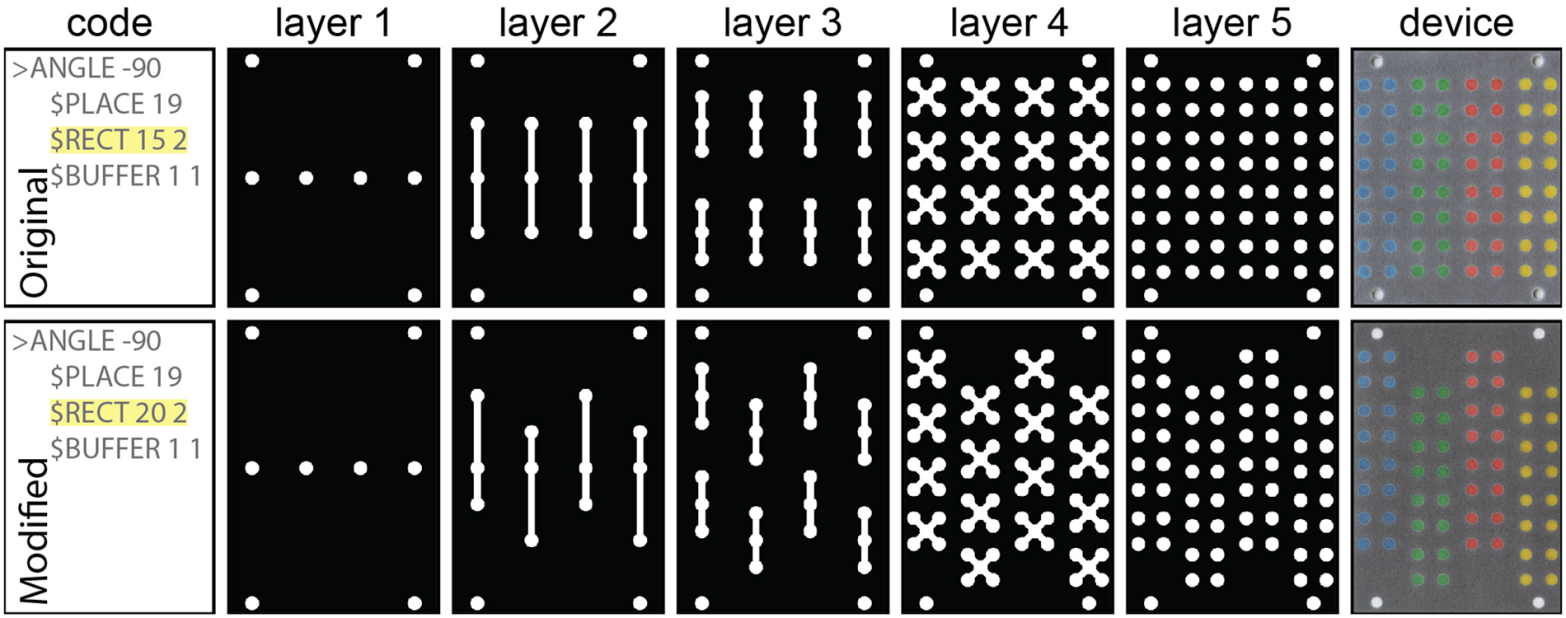
Use of AutoPAD to design a multilayer device that splits four samples into sixty-four test zones. The left panel displays a section of code used to create the design, where the command to be modified is highlighted in yellow. The middle panels (layers 1–5) display the AutoPAD outputs (as PNG files) of the full device design. The right panel is an image of a paper-based device fabricated from the AutoPAD design with dye added to each of the sample introduction zones. The top and bottom rows illustrate original and modified designs. Cut layers for adhesives were also generated by AutoPAD, but those images are not shown for clarity. Adapted from Martinez *et al*.^13^.

We recreated a three-dimensional sample splitter^13^ in order to demonstrate AutoPAD’s ability to produce complex patterns that require precise alignment between multiple layers of paper. In total, this device design comprises 364 individual elements distributed across 9 layers of patterned materials: 4 objects on layer 1 (paper), 4 objects on layer 2 (adhesive), 20 objects on layer 3 (paper), 8 objects on layer 4 (adhesive), 40 objects on layer 5 (paper), 16 objects on layer 6 (adhesive), 144 objects on layer 7 (paper), 64 objects on layer 8 (adhesive), and 64 objects on layer 9 (paper). An automated approach to preparing such a design would limit operational errors (e.g., leaks or failures) caused by any misalignment between layers that could result from a manual design process. We present the layers of the three-dimensional sample splitter design in Fig. 4 (Case Study 5).

By using this referencing approach, splitting device designs with altered dimensions (Fig. 4) require only minor alterations to the script rather than a complete restructuring and repositioning of all device design elements across multiple layers. The simple modifications that we made to Layer 2 of this device caused numerous automatic changes in subsequent layers of the device. For example, changing the length of 1 rectangular zone in Layer 2 of this device causes the positions of 43 additional design elements—32 from paper layers and 11 from adhesive layers—to also change. When we subsequently modify the length of all 8 rectangular zones in Layer 2, which takes just 2–3 minutes in AutoPAD, 344 design elements are automatically updated to yield a properly aligned, functioning device design. In a commercial software like Adobe Illustrator, this type of design modification would require a significant time investment (ca. hours), as it requires repositioning of 95% of all device elements.

### Case Study 6. Origami Devices

An additional function of AutoPAD is the ability to construct origami-styled devices^14,16,40^. Because they are manufactured using a single layer of folded paper, components of origami designs require very careful spatial organization and alignment in order to ensure that proper contact is made between all zones to complete the desired, intact network of hydrophilic channels. Origami devices demonstrate the use of Combined-Layer commands, which is a functionality of the AutoPAD Interpreter that allows for multiple layer designs to be tiled into a sheet. The arrangement of individual tiles can be controlled through rotation, reflection, and positioning commands to create the desired array of zones. To demonstrate these concepts, we recreated the first demonstration of the use of origami to assemble three-dimensional paper-based microfluidic devices (Supplementary Fig. S4, Case Study 6)^14^. This design required the use of only three layers (seen in the first row), which were then rotated in order to complete the grid. Similar to the previous case studies, the origami design is also easy to refactor with changes to only a single parameter due to AutoPAD’s ability to perform simple arithmetic. Note that it is possible that some altered designs may not fold in a manner that creates functional fluidic connections; future versions of AutoPAD that are driven by user need could offer more sophisticated folding-alignment prediction capabilities.

### Time Investment to Create Designs

Once a user has become familiar with AutoPAD, device designs can be created quickly. For example, the sample splitter, microzone plate, 2DPN, and three-dimensional immunoassay devices can each be created in ca. 5–10 minutes using our software. Considering that this period of time also includes the creation of fully tiled 8.5 in. × 11 in. sheets and any necessary cutting masks (e.g., for layers of adhesive to affix multiple layers of paper), AutoPAD represents an improvement over conventional methods that rely on drawing and aligning individual elements. In particular, a substantial time savings is obtained when designing three-dimensional immunoassays, which can take up to 1 hr. to design in a conventional software like Adobe Illustrator. Our immunoassay platform is a multilayer device—comprising five layers of patterned paper and five layers of patterned adhesive—that is tiled over a full, printable sheet^17^. Modifications to our case study designs can be achieved quickly (ca. 2 minutes per design) by changing only 1–3 parameters in their designs (e.g., Supplementary Fig. S3). Due to their complexity, we did not observe time gains when creating the initial versions of the multilayer sample splitter or origami devices (ca. 30 minutes). However, due to the speed with which AutoPAD refactors designs automatically, users can generate subsequent prototype devices with minimal effort. For example, modifications to these complex devices are produced relatively quickly (ca. 5 minutes) in AutoPAD by changing only a few parameters in the Interpreter (Fig. 4 and Supplementary Fig. S4). In Adobe Illustrator, the multi-splitter design would take hours to design, with a similar time investment required to realign layers for the smallest design modifications. The process of using AutoPAD, therefore, differs considerably from current methods that would require the user to either realign numerous components across multiple layers of a device or, more likely, initiate an entirely new design.

## Conclusions

In this manuscript, we have described the development of AutoPAD—the first software program created specifically to enable the design of paper-based microfluidic devices. We used AutoPAD to recreate several literature examples of paper-based microfluidic devices (e.g., paper microzone plates^43^ and three-dimensional immunoassays^17^) and demonstrated how AutoPAD could be used to generate design modifications with minimal user effort. We expect that the ease and rapid pace with which designs can be modified will aid users in the processes of prototyping devices and optimizing the performance of assays, and ultimately lead to device designs with improved manufacturability and reproducibility. We anticipate that automatic zone alignment and refactoring capabilities—functions that we programmed to be inherent to AutoPAD—will aid in the exploration of more complex design structures^55^, and facilitate the broader use of advanced design features (e.g., paper-based valves^56^ or sliding strips^57)^. However, we acknowledge that there is a significant learning curve to becoming adept with AutoPAD. Potential users must, as with any other new program, become accustomed to the layout, syntax, and operation of our software; in particular, thinking of devices as networks of connected shapes may be somewhat foreign to users of conventional software platforms that rely on manually drawing and aligning geometric shapes. We believe that the potential benefits that result from using AutoPAD to design paper-based devices—particularly three-dimensional devices that comprise intricate microfluidic networks—justify this investment of time. To support the adoption of AutoPAD and facilitate the training process, we have created a series of documentation and video tutorials that we include as part of the Supplementary Information. We believe AutoPAD will lower the barrier of entry into the field for researchers with limited access to costly, commercial design software and ultimately facilitate the development of the next generation of point-of-care diagnostic assays. To support these goals, we provide AutoPAD and its source code free-of-charge, in an open-source and cross-platform format^42^.

Not only do we encourage others to use AutoPAD, but also to contribute to its continued development, improvement, and evolution as an enabling research tool for the paper-based microfluidics community. For example, future versions of AutoPAD could be designed to enable the visualization of the alignment of zones between layers in assembled 3D devices. Additionally, click-and-drag features would improve intuition and recruit a user base that has experience in other graphic-based design software—the learning curve could be substantially mitigated if users could change the design code automatically by moving device elements within the preview window. Finally, incorporating pre-loaded designs, or templates, into the software could also be very helpful for those who are new to designing paper-based devices. While there are opportunities to expand on the preliminary features of AutoPAD, the first version of this software has the potential to simplify the prototyping process for paper-based microfluidic devices, enable accessibility and collaboration within the field, and facilitate the development of user-defined, application-specific software tools that will streamline the development of low-cost diagnostics.

### Data availability

All data generated or analyzed during this study are included in this published article (and its Supplementary Information files).

## Electronic supplementary material

Materials and Methods

Glossary

Introduction to the Tree Interface

Getting Started with AutoPAD
