# Supplementary material for: An Open Software Platform for the Automated Design of Paper-Based Microfluidic Devices
Source: Sci Rep. 2017 Nov 24;7:16224. doi: 10.1038/s41598-017-16542-8 (PMC5701164; doi:10.1038/s41598-017-16542-8)
Supplement: Supplementary file 1 — Materials and Methods [file 41598_2017_16542_MOESM1_ESM.pdf]

An Open Software Platform for the Automated Design of Paper-Based Microfluidic Devices

Nicholas S. DeChiara, Daniel J. Wilson, and Charles R. Mace*

Department of Chemistry, Tufts University, 62 Talbot Avenue, Medford, MA 02155 USA

*Corresponding author: charles.mace@tufts.edu

Supplementary Information 1: Materials and Methods (12 pp)

Writing the AutoPAD software

We chose Java to create AutoPAD because, as a cross-platform programming language, the same code can run on Windows, Apple, Linux, and even Android computers with little to no modification required. Using Java to create AutoPAD allows our codebase to be broadly accessible to other users to modify, re-compile, and share freely as new applications are developed by the community. We chose the SWT library to provide graphical user interface support, as it is open-source, cross-platform, and available under the Eclipse Public License, which allows for free use of this library. We used PDFBox to generate PDF files of device designs, for similar reasons. We performed all programming in the Eclipse IDE (Integrated Development Environment) and compiled all program files in Eclipse, which is another free software package. This choice, though, should have little impact on future use or modification of the AutoPAD code, as it can be loaded into any Java-compatible development environment.

We divided the software itself into two packages: an Interpreter and an Interface. The Interpreter was built first. It can read scripts and create images out of those scripts. The Interface was built after the Interpreter and provides a graphical user interface, which is used to write scripts and access the features of the Interpreter. At the most basic level, all designs made with this software exist as scripts formatted using a scripting language devised specifically for AutoPAD. The scripting language can create explicitly-defined shapes and place them exactly where intended without the possibility of small and difficult-to-notice human errors associated with manual drawing, which may ultimately lead to challenges in the manufacture of devices or even failure of assays. The architecture of designs in this language is a relative-node system, which means that design elements are divided into discrete objects. Instead of having absolute positions in the design space, these objects are positioned relative to other objects. We took this

approach because the important factor in device design is the distance between objects, not their positions relative to a common origin. Relative positioning also aids in refactoring, as changing the distance between two objects results in the automatic adjustment of all the objects attached to those objects, and users are therefore not required to make corrections manually (a potentially laborious and error-prone process). The design of our program enables simple and rapid design of complex, multilayered, paper-based microfluidic devices.

Installing and Using the AutoPAD Software

While we refer readers to three additional supporting documents—*Glossary*, *Introduction to the Tree Interface*, and *Getting Started with AutoPAD*—that accompany this manuscript (available freely online as **Supplementary Information**) for a comprehensive description of our software's core functions, as well as the source code, program builds, and case study files hosted on our laboratory GitHub repository (https://github.com/MaceLab/AutoPAD), there are three operations that are fundamental to beginning with AutoPAD: (i) installing the software, (ii) saving device designs, (iii) opening saved design files.

Installing AutoPAD in Windows and OSX

Installing AutoPAD is a short process. First, select the version that corresponds to your operating system and download the corresponding Zip file. Extract the Zip file to any desired location on your computer. AutoPAD should now be ready to use. It is important to note that the files and folders comprising this extracted folder each have an explicit purpose, and AutoPAD may not run correctly if any of this folder's contents are modified or deleted.

On Windows, you can run AutoPAD by double-clicking the included BAT files. On Mac operating systems, you can run AutoPAD by clicking on the included COMMAND files. Apple's default security settings may block you from opening AutoPAD, as it was not created by an Apple identified developer. These settings can be adjusted from the "Security & Privacy" window under the "General" tab when you attempt to open AutoPAD. To run AutoPAD, you must have Java installed on your computer. If you already have Java installed on your computer, you may need to update it for AutoPAD to function properly. On Windows and OSX, this can be accomplished simply by navigating to https://java.com/en/download/ and following the steps on that website.

The 32-bit version of AutoPAD built for Windows will run on both 32- and 64-bit Windows machines. To determine whether you should download 32 or 64-bit AutoPAD for Apple computers, you must determine the specifications of your operating system. On OSX computers, open the Apple menu and select "About This Mac". Select the "More Info" option and open the "Hardware Section". If your "Processor Name" is "Intel Core Solo" or "Intel Core Duo", then your computer is 32-bit. Otherwise, your computer is 64-bit.

Saving Device Design Files

To save a design file (as a text file), click "File" and "Save As". Next, select the location you would like to save the script to, and then click "Save". Selecting "File" and "Save" will save this file to the same location until a new location is specified with "Save As." To export a design file (as a series of PNG images and tiled PDFs), click "Build" and "Target Folder" to select the desired destination of the PNG and PDF files. Next, Click "Build" and "Run" to generate the PNG and PDF files in the selected location. Selecting "Build" and "Run As" will allow you to

generate a TXT script file in a desired location, while also generating PDFs and PNGs in a previously selected target folder location. By default, running a script without selecting a target folder will save the output files to the "out" folder inside of the AutoPAD software folder.

Opening Saved Design Files

To open a design file, open the AutoPAD program first. Next, click "File" and "Open". Next, select the location of the file you wish to open. Select the file and click "Open". This allows a user to return to a previously saved or shared design, facilitating the sharing of design files among multiple users. Design text files may also be run directly from the Interpreter, which can bypass the need to open the Tree Interface.

Materials and Methods

Case Studies

We produced sample devices using images from the AutoPAD software. For each design, we patterned each layer on 8.5 in. by 11 in. Whatman 4 chromatography paper using a wax printer (Xerox ColorQube 8580). To define hydrophobic barriers in the paper layers, we melted the wax in a 150 °C oven. We assembled our devices using double-sided adhesive (FLEXcon) cut by a robotic plotter (Graphtec CE6000) using patterns generated in AutoPAD. Layers of nitrocellulose, polyethersulfone, and paper were patterned using a laser cutter (Trotec). Devices were assembled according to a previously reported protocol for the fabrication of paper-based devices.[1] Sample devices were run using McCormick brand food coloring solutions.

Vectorizing Cut Files

AutoPAD outputs cut patterns as PNG files, but our knife plotter and laser cutter require vectorized paths for use. In order to use our designs with our knife plotter or laser cutter, we had to vectorize the images of our cut patterns using Adobe Illustrator. To vectorize these designs we followed a general protocol that consisted of the following steps: (i) release any clipping masks from the original design, (ii) convert colors to grayscale, (iii) rasterize (at 300 dpi in grayscale color model), (iv) use the Image Trace tool (using Line Art preset or custom settings) to trace the contours of the AutoPAD design, and (iv) use the Expand button to generate vectors from the trace. After vectorization, the design elements can be ungrouped (and compound paths released) to allow for excess design elements to be deleted before cutting. After this process is complete, the vectorized design paths can be cut on the knife plotter or laser cutter.

Figure S1. Designing paper microzone plates. The original design from Carrilho et al.[2] replicated the geometry of a 96-well plate patterned into chromatography paper. We redrew the original design using AutoPAD and generated a working paper microzone plate from our design. Simple modifications of our design (sections of code highlighted in yellow), in which we connected adjacent wells or changed the shape of each well, were made rapidly using the referencing features of AutoPAD. These designs were also patterned in chromatography paper and made into working devices.

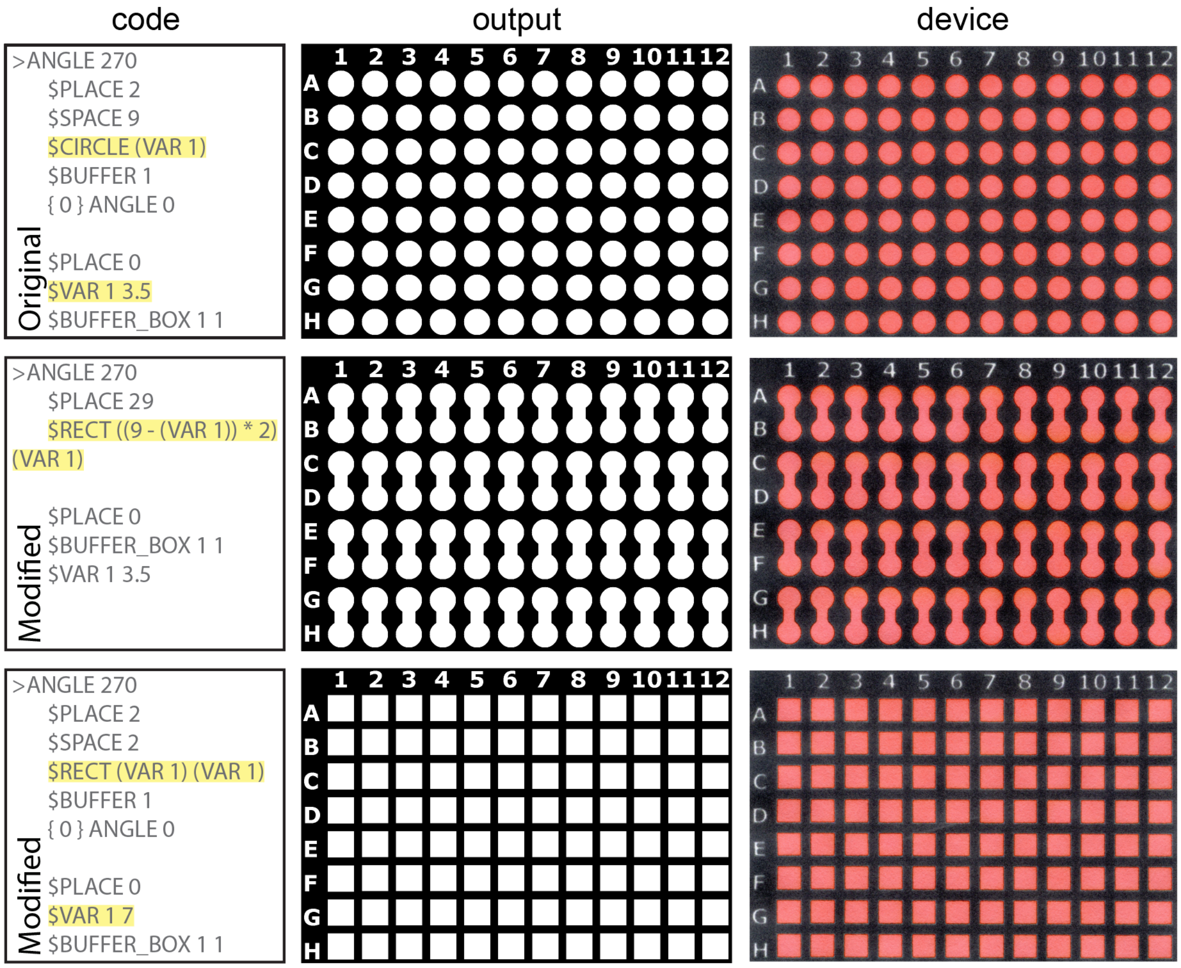

Figure S2. Designing two-dimensional paper networks (2DPN). The original design from Fu et al.[3] comprised laser cut patterns of membrane used to visualize and measure flow in porous matrices. We redrew the original design using AutoPAD and generated laser cutter-compatible files from our design. A simple modification of our design, in which we lengthened the device and added an inlet (section of displayed code copied 3x instead of 2x), was made rapidly using the node connectivity of AutoPAD. These designs were laser cut from chromatography paper, polyethersulfone membrane (PS), and backed nitrocellulose membrane (NC). Additionally, the cut designs generated by AutoPAD can be used with robotic knife plotters, as demonstrated using a piece of backed double-sided adhesive.

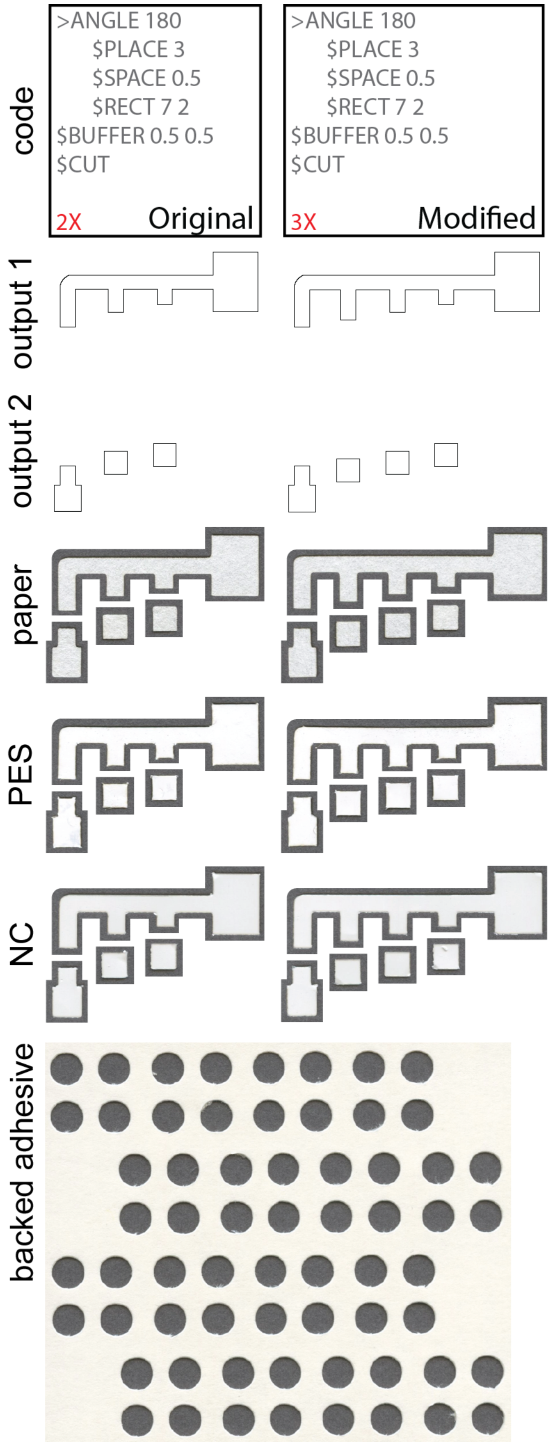
code
output 1
output 2
paper
PES
NC
backed adhesive
>ANGLE 180
$PLACE 3
$SPACE 0.5
$RECT 7 2
$BUFFER 0.5 0.5
$CUT
2X
Original
>ANGLE 180
$PLACE 3
$SPACE 0.5
$RECT 7 2
$BUFFER 0.5 0.5
$CUT
3X
Modified

Figure S3. Designing three-dimensional paper-based immunoassays. The original design from Schonhorn et al.[4] directed the applied sample through a multilayered paper network, which contained stored reagents, to complete an immunoassay. We redrew the original designs using AutoPAD, and patterned layers of chromatography paper with these designs to create a working device. We made a simple adjustment to the device geometry by changing the length of the incubation pathway in layer 3 (section of code highlighted in yellow), which resulted in automated refactoring of every subsequent layer in the device. Cut layers for adhesives were also generated by AutoPAD, but those images are not shown for clarity. Because the hole geometry in each cut layer here is the same, cut layer designs for this device can be duplicated for convenience. The detection layer of the devices generated from the original and modified designs was treated with dye. After assembly of the multilayer devices, each device was run with water, which washed the dye away to demonstrate that each device produced a complete microfluidic network.

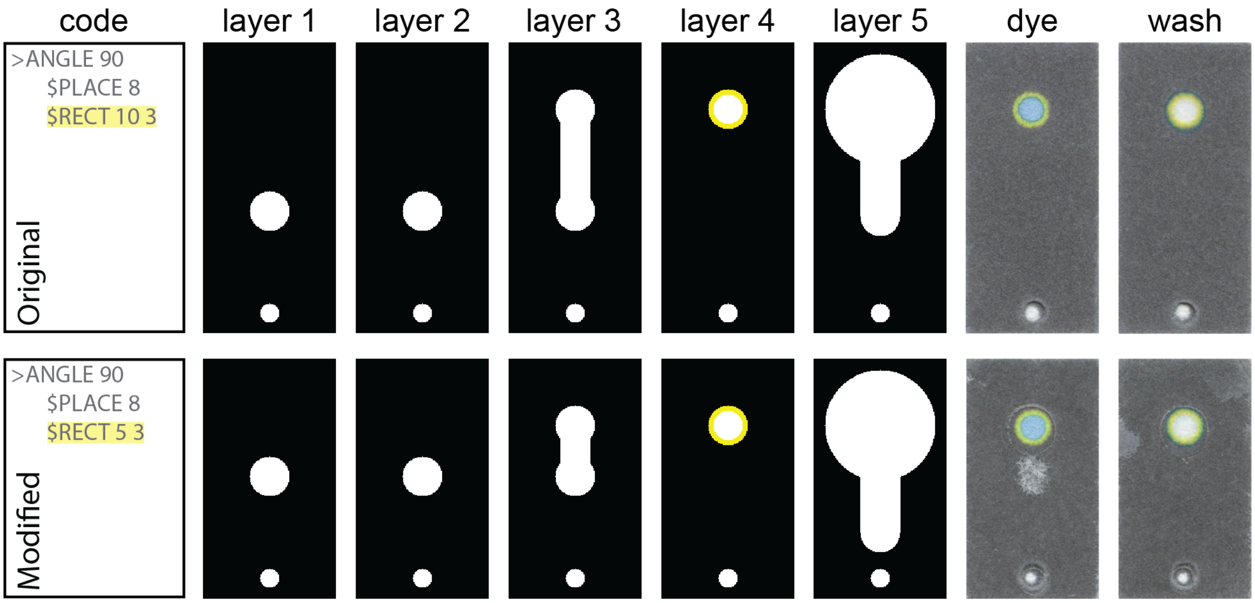

Figure S4. Designing origami devices. The original design from Liu et al.[5] directed the applied sample into several testing zones within multiple layers of folded paper. We redrew the original origami design using AutoPAD. With two simple code modifications (sections of code highlighted in yellow), we were able to enlarge the spacing among zones, changing the scale of the entire device. We patterned both of these designs in chromatography paper, folded the devices according to the original protocol, and ran them using solutions of food coloring.

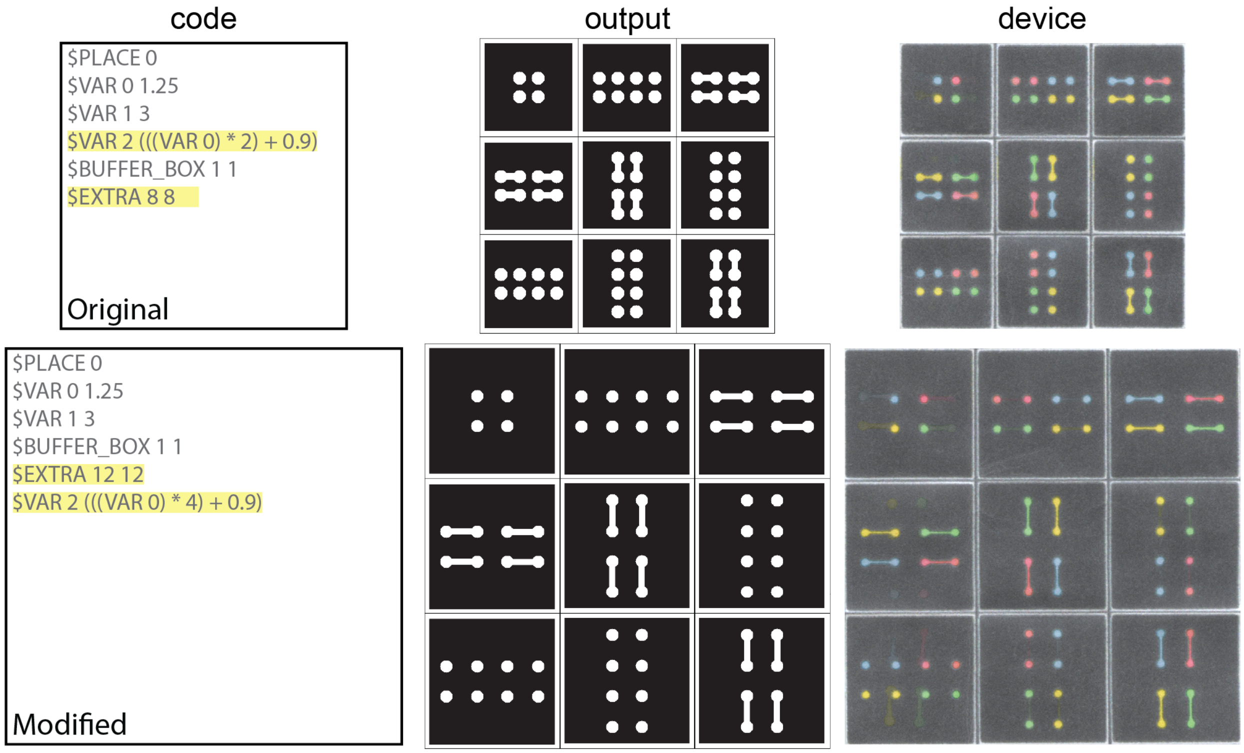

References

1. Fernandes, S.C., Wilson, D.J. & Mace, C.R. Fabrication of three-dimensional paper-based microfluidic devices for immunoassays. *Journal of Visualized Experiments* **121**, e55287, doi: 10.3791/55287 (2017).
2. Carrilho, E., Phillips, S.T., Vella, S.J., Martinez, A.W. & Whitesides, G.M. Paper microzone plates. *Analytical Chemistry* **81**, 5990–5998, doi: 10.1021/ac900847g (2009).
3. Fu, E., Liang, T., Spicar-Mihalic, P., Houghtaling, J., Ramachandran, S. & Yager, P. Two-dimensional paper network format that enables simple multistep assays for use in low-resource settings in the context of malaria antigen detection. *Analytical Chemistry* **84**, 4574–4579, doi: 10.1021/ac300689s (2012).
4. Schonhorn, J.E., Fernandes, S.C., Rajaratnam, A., Deraney, R.N., Rolland, J.P. & Mace, C.R. A device architecture for three-dimensional, patterned paper immunoassays. *Lab on a Chip* **14**, 4653–4658, doi: 10.1039/c4lc00876f (2014).
5. Liu, H. & Crooks, R.M. Three-dimensional paper microfluidic devices assembled using the principles of origami. *Journal of the American Chemical Society* **133**, 17564–17566, doi: 10.1021/ja2071779 (2011).
