# Supplementary material for: An Open Software Platform for the Automated Design of Paper-Based Microfluidic Devices
Source: Sci Rep. 2017 Nov 24;7:16224. doi: 10.1038/s41598-017-16542-8 (PMC5701164; doi:10.1038/s41598-017-16542-8)
Supplement: Supplementary file 2 — Glossary [file 41598_2017_16542_MOESM2_ESM.pdf]

An Open Software Platform for the Automated Design of Paper-Based Microfluidic Devices

Nicholas S. DeChiara, Daniel J. Wilson, and Charles R. Mace*

Department of Chemistry, Tufts University, 62 Talbot Avenue, Medford, MA 02155 USA

*Corresponding author: charles.mace@tufts.edu

Supplementary Information 2: Glossary (16 pp)

Glossary

Note: While commands are listed in all caps, none of them are case-sensitive.

Header Commands

Header commands begin with an angle bracket (the '>' character) and indicate directionality; the '>' can be read as "go to", accordingly. Header commands denote movement to a different node, either new or existing. To this end, we will consider header nodes in two categories: new and previous.

New

- *Directions*
  - Angle: "ANGLE" followed by a number indicates a new node at the current node's position, going towards the specified angle. Nodes created via. angle inherit the layer of their parent node.
    - Ex. ">ANGLE 30" makes a new node towards the 30 degree mark.
  - Cardinal: "LEFT", "RIGHT", "UP" and "DOWN" are automatically translated into Angle nodes (0, 180, 90 and 270, respectively).
    - Ex. ">RIGHT" makes a new node towards the 180 degree mark.
- *Layers*
  - Layer or Z: "LAYER" or "Z" followed by a number indicates a new node on the specified layer, at the current node's position. Nodes created via layer inherit the angle of their parent node.
    - Ex. >LAYER 3 makes a new node on the third layer.
    - Note: "LAYER" and "Z" are functionally identical.

Previous

- *Places*
  - Nodes marked via "$PLACE" and a number can be returned to by referencing that number. ">" and a number sets the current node in this fashion. Each script starts with ">0", as the default starting node is always place 0.
    - Ex. ">3" returns to the node that has "$PLACE 3".
    - Note: only places 1-1248 are accessible.
- *Temporary Places*
  - Nodes marked via "$TEMP" and a number can be returned to in the same fashion as places can be returned to. ">TEMP" and a number is used for this process.

Temporary places are functionally the same as places but are intended to be reused, whereas places are intended to be set once and not changed.

        - Ex. “>TEMP 4” returns to the most recent node that has “$TEMP 4”.
        - Note: only temp places 0-1248 are accessible.

- *Back*
    - A list of the most recently visited nodes is kept and known as the “previous nodes list”. This list can be accessed via the “>BACK” command. “>BACK” and a number goes back that many spaces on the previous nodes list and fetches that node.
        - Ex. “>BACK 1” returns to the previous node
        - Ex. “>BACK 2” returns to the node before that
        - Ex. “>BACK 0” gives the current node
        - Note: only the past 64 nodes are kept, so this command will not function beyond that.
- *Raw ID*
    - A list of all nodes in the device is kept and known as the “allnodes” list. This list can be accessed in the same fashion as the places list. “>” followed by a node id will make that node the current node. Node ids begin at 10000 (equivalent to place 0, the default first node) and go up as new nodes are added (ex. the very second node after place 0 will be 10001).
        - Ex. “>10009” returns to the 9th node created beyond the default node.

Node Property Commands

Node property commands are the pillars of scripting with AutoPAD. These commands define all of the important properties of a node, except for layer and angle which are defined by header commands (or inherited). When a node property command is used, it is attached to the most recent node.

Shape

- *Rectangles*
    - “$RECT” followed by one or two numbers makes the node a rectangle. The first number is the length of the rectangle (i.e. in the direction of the node’s angle) and the second number is the width of the rectangle (perpendicular to the node’s angle). If only the length is given, the width will be assumed to be the same (i.e. a square).
        - Ex. “$RECT 4 2” makes a rectangle that is 4 long and 2 wide.
        - Ex. “$RECT 3” makes a square that is 3 long and 3 wide.
- *Corners*
    - “$CORNER” followed by three numbers makes the node a triangle. The first number is the base of the triangle, the second is the length of the leftmost side, and the third

number is the bottom-left angle. If the angle is negative, the second number is then the length of the rightmost side and the angle becomes the bottom-right angle. Essentially, this command takes the length of two sides and the angle between them.

    - Ex. “$CORNER 2 1 90” makes a right-triangle with a base of 2 and a height of 1.
    - Ex. “$CORNER 1 1 30” makes a triangle where two sides are each 1 long and the angle between those sides is 30 degrees.
    - Ex. “$CORNER 1 2 -45” makes a triangle whose base is 1 long and that has a side that is 2 long, where the angle between those two sides is 45 degrees, and the angle is on the opposite side as the prior example.
  - “$CORNER_CURVE” followed by a number makes this node have a curved third side, the amount of curve depending on the magnitude of the number given. By default, each corner has a curve weight of 1.0, and a higher curve weight will create a concave curve while a lower curve weight will create a convex curve.
    - Ex. “$CORNER_CURVE 1.5” makes the triangle on this node have a concave curved third side.
- *Circles*
  - “$CIRCLE” followed by one or two numbers makes the node a circle. The first number is the radius of the circle and the second number is the optional inner radius of the circle. If the inner radius is non-zero, the circle will become a disk.
    - Ex. “$CIRCLE 4” makes a circle of radius 4
    - Ex. “$CIRCLE 3 1” makes a circle of radius 3 with a circle of radius 1 sized hole in it.
  - “$DISK” is functionally the same as “$CIRCLE”
  - “$CIRCLE_INNER” and a number sets only the inner radius of a circle.
- *Polygons*
  - “$POLY” establishes this node as a polygon. Polygons are formed out of a list of vertices, and any simple polygon (i.e. whose edges do not intersect each other) is viable.
    - Ex. “$POLY” sets this node as a polygon.
  - “$VERTEX”, “$VERT”, “$VEX” or “$VX” sets a vertex. This command is followed by up to 5 numbers, 2 of which are necessary: X, Y, Type, Buffer, Extra. X and Y are the position of the vertex relative to the node’s center (ex. 0,0 is at the node, 1,0 is forward in the direction of the node’s angle, etc). Type is the type of line connecting this vertex to the next, where 0 is a straight line, 1 is curved concave down and 2 is curved concave up. Buffer is the amount of buffer space at this vertex. Extra is the amount of extra white space at this vertex.
    - Ex. “$VERTEX 0 1” “$VERTEX 2 1” “$VERTEX 2 -1” “$VERTEX 0 -1” defines a square of size 2x2. (note each command must have its own line)

        - Ex. “$VERTEX 0 0 1 2 4” defines a vertex at 0,0 (the node position) which curves up, has buffer of radius 2, and extra space of radius 4.
    - “$CURVE_WEIGHT” and a number sets the curvature of the last vertex, if that vertex is type 1 or 2. This value is 1.0 by default; 0.0 would produce a straight line, and the values in-between would be more straight or more curved depending on how far from 0 or 1 they are. Values greater than 1 produce more curved shapes, while values less than 0 invert the concavity of the curve.
        - Ex. “$CURVE_WEIGHT 0.5” makes the last vertex’s curve be half as curvy and more straight.
        - Ex. “$CURVE_WEIGHT 2” makes the last vertex’s curve bulge out further.
    - “ROT_CENTER” followed by two numbers sets the center of rotation for this polygon.
        - Ex. “ROT_CENTER 0 2” sets the polygon to rotate around the point 0,2 from the node center.
        - Note: unlike other points, this point is not relative to the node’s angle and is instead absolute (i.e. for a node of angle 0).
    - “$OUTLINE” sets this polygon to not be filled in and to only be an outline.
- *Text*
    - “$TEXT” and some text sets this node to have text attached to it.
        - Ex. “$TEXT positive (+)” writes “positive (+)” on this node.
    - “$TEXT_SIZE” and a number specifies the font size of the text on this node. As an important note, font size by default is equivalent to the $FILL conversion size, and this parameter is how many times larger or smaller the font size is than that conversion size. For instance, if the $FILL conversion is 72, then the default font size is 72pt. In that same scenario, if “$TEXT_SIZE 0.5” was used, the font size at that node would be 36pt. This system is in place to ensure that text is the same relative size regardless of which $FILL conversion the image is generated at.
        - Ex. “$TEXT_SIZE 2.5” would change the font size to be 2.5x the $FILL conversion size.
    - “$TEXT_FONT” and a font specifies the font on this node. This should function for any font installed on the active computer, so long as the name is specified correctly. The default font is Verdana.
        - Ex. “$TEXT_FONT Arial” would switch the font to Arial.

Property

- *Place*
    - “$PLACE” followed by a number gives this node a place marker so that it can be returned to via the “>” command.
        - Ex. “$PLACE 3” sets this node to be the third place.

        - Note: only places 1-1248 are accessible.
- *Temp*
    - “$TEMP” followed by a number gives this node a temporary marker (overwriting existing markers of the same number) so that it can be returned to via the “>TEMP” command.
        - Ex. “$TEMP 5” sets this node to be the fifth temporary marker.
        - Note: only markers 0-1248 are accessible.
- *Color*
    - “$COLOR” followed by three numbers sets the RGB color value of this node. “$COLOR” followed by the name or first three letters of a common color also works. For those unfamiliar with 24-bit RGB coloring, a color is defined by three integers which each range from 0 to 255 (because an unsigned byte can store 256 values, and in this system each pixel is stored as three bytes) and correspond to the red, green and blue content of the color, respectively. For convenience, it is also possible to encode colors as numbers ranging from 0.0 to 1.0 (which will automatically be converted to the 255 system, for instance 0.5 becomes 127); if no color value exceeds 1.0, this convention will automatically be used.
        - Ex. “$COLOR 255 0 0” sets the color to red.
        - Ex. “$COLOR YEL” sets the color to yellow.
        - Ex. “$COLOR PURPLE” sets the color to purple.
        - Ex. “$COLOR 0.5 0.5 0.5” sets the color to gray.
        - Note: the color of a node is white by default.
        - Note: the list of color shortcuts is as follows:
            - WHI or WHITE or “255 255 255” “1 1 1”
            - BLA or BLACK or “0 0 0” “0 0 0”
            - BLU or BLUE or “0 0 255” “0 0 1”
            - RED or “255 0 0” “1 0 0”
            - GRE or GREEN or “0 255 0” “0 1 0”
            - ORA or ORANGE or “255 125 0” “1 0.49 0”
            - PUR or PURPLE or “255 0 255” “1 0 1”
            - YEL or YELLOW or “255 255 0” “1 1 0”
            - GRA or GRAY or “125 125 125” “0.49 0.49 0.49”
            - TEA or TEAL or “0 255 255” “0 1 1”
- *Space*
    - “$SPACE” followed by a number sets the distance of this node from the last.
        - Ex. “$SPACE 1.5” sets a distance of 1.5 from the last node.
        - Note: negative values work, and will make shapes overlap.
- *Perpendicular Space*
    - “$PERPSPACE” followed by a number sets the distance of this node from the last in the axis perpendicular to their axis of alignment.

        - Ex. “$PERPSPACE 2” sets a distance of 2 at a right angle from the last node.
        - Note: negative values work, and will make shapes travel in the other direction on the perpendicular axis.

- *Buffer*
    - “$BUFFER” followed by one or two numbers establishes the amount of black space surrounding nodes. The first number is the length of buffer (i.e. in the direction of the node), and the second number is the width of buffer (perpendicular to the direction of the node). If only the length is given, the width will default to be the same value. For circles, giving only one number makes the buffer circular. Giving two makes the buffer a box. For polygons, only one number should be given.
        - Ex. “BUFFER 2 4” makes a buffer that is 2 long and 4 wide.
        - Ex. “BUFFER 1” makes a buffer that is 1 long and 1 wide.
- *Precise*
    - “$PRECISE” sets a node to have ‘precise’ spacing relative to its neighbor nodes. Normally, a node starts at the position of the prior node. For instance, if the last node was a circle, the new node would start in the center of that circle. However, if ‘precise’ is enabled, the new node would instead start on the edge of the circle.
        - Ex. “$PRECISE” enables ‘precise’ mode for this node
        - Note: “precise” mode is inconsistent with polygons.
- *Cut*
    - “$CUT” marks this node to have its outline included in the cut layer. If any node on a layer has ‘cut’ enabled, an additional cut layer image will be generated for that layer which has only the outlines of the nodes set to be cut.
        - Ex. “$CUT” marks this node to be cut.

Global

Global commands should only be used once per script, and should usually go at the end of the script, attached to the default first node (place 0).

- *Fill*
    - “$FILL” is one of the most important commands in the script. It must be called at the end of each script, off of the place 0 node. “$FILL” is followed by a number and some text, and triggers the creation of the image files associated with this script, which will be scaled up by a factor of the number and saved with the filename of the text.
        - Ex. “$FILL 20 test” scales so that a value of “1” is translates to 20 pixels, and saves the images as “20 test_#.png”, where # is the layer number. For example, “$RECT 1 1” would make a 20x20 pixel rectangle under this setting.

        - The fill factor should correspond to the PPI (pixels per inch) of the printer that will be used. For instance, a PPI of 300 means that 300 pixels form an inch, and "$FILL 300 test" would make "$RECT 1 1" give a 1in by 1in rectangle.
- *Cut*
    - "$CUT_OVERLAP" enables overlapping cut outlines. If this command is not used, overlapping edges will be removed on the cut layers.
        - Ex. "CUT_OVERLAP" enables cut overlapping.
- *Buffer*
    - "$BUFFER_BOX" disables snap-to-shape buffering and replaces it with a single box of buffering. "$BUFFER_BOX" is followed by two numbers, x and y extra, which determine how much extra white space is on each side.
        - Ex. "$BUFFER_BOX 2 3" enables buffer boxing and leaves 2 units of space on either side, and 3 units of space on top and bottom.
- *PDF*
    - "$PDF_OFF" disables PDF generation. By default, each image produced will be automatically put into a PDF file. However, when this command is used, this feature is disabled.
    - "$PDF_SIZE_IN" followed by two numbers changes the size of the PDF files that are generated, where the first number is the width of the page and the second number is the height of the page, in inches. By default, standard letter-sized PDFs are generated.
        - Ex. "$PDF_SIZE_IN 4.5 6" changes the PDF size to a 4.5 x 6 inch size.
    - "$PDF_SIZE_CM" followed by two numbers functions exactly as "$PDF_SIZE_IN", except the width and height are in centimeters instead.
        - Ex. "$PDF_SIZE_CM 10.5 14.8" changes the PDF size to a 10.5 x 14.8 cm (A6) size.
    - "$PDF_MARGIN_IN" followed by two numbers changes the PDF edge margins that are left as white space, where the first number is the width of the horizontal margins and the second number is the height of the vertical margins, in inches. By default, half-inch margins are used.
        - Ex. "$PDF_MARGIN_IN 0.1 2" changes the page margins to be a tenth of an inch on the right and left sides and 2 inches on the top and bottom of the page.
    - "$PDF_MARGIN_CM" followed by two numbers functions exactly as "$PDF_MARGIN_IN", except the width and height are in centimeters instead.
        - Ex. "$PDF_MARGIN_CM 3 1" changes the page margins to be 3 centimeters horizontally and 1 centimeter vertically.
- *Debug*
    - "$DEBUG" is a command that makes the compiler output more information, which can help when hunting down errors. This should be called first in a script.

Functions

- *Variables*
  - “$VAR” followed by two numbers sets the value of a variable. The variable ID is the first number, the second number is the variable’s value. Variables are used as reference values which can be brought back when desired. See the section on Value Parsing for more on this.
    - Ex. “$VAR 1 23.1” sets variable 1 equal to 23.1
    - Note: only variables 0-63 are accessible.
- *References*
  - “{ name }” calls back a reference with the specified name or number to be replicated. This command can be followed by “x” and a number to repeat this command that number of times. This command can also be followed by “ANGLE” and a number to add that number to all of the angles in this reference. Finally, this command can also be then followed by up to 9 numbers to set variables 0-8 to those numbers.
    - Ex. “{ 11 } x 3” calls back reference 11 and repeats it 3 times
    - Ex. “{ quicktriangle } ANGLE 10” calls back reference “quicktriangle” and moves all of its angles over 10 degrees.
    - Ex. “{ 8 } x 2 ANGLE 30” calls back reference 8, repeats it twice, and moves all of its angles by 30 degrees.
    - Ex. “{ 0 } x 3 ANGLE 15 VARS 1 2.5 4” calls back reference 0, repeats it 3 times, adjusts its angles by 15 degrees, and sets variable 0 = 1, variable 1 = 2.5, and variable 2 = 4
    - Ex. “{ testref } VARS 3 9 55.5” calls back reference “testref” and sets variable 0 = 3, variable 1 = 9, and variable 2 = 55.5.
    - Note: when called, the code from the reference is essentially copied and pasted into the position where the “{ }” call is.

Value Parsing

All number values in scripts are parsed via the value parser, which has a specific format: each operation is contained within its own pair of parentheses, and there must be a space between operators and numbers. Operations within more parentheses are performed first.

Input	Output	Input	Output	Input	Output
(1 + 1)	2	(2 ^ 3)	8	(8 % 2)	0
((2 * 3) - 1)	5	(4 ^ 0.5)	2	(COS 0)	1
((2 + 1) * (2 + 3))	15	(10 / 2)	5	(FLOOR 1.1)	1

- *Addition*
  - “(# + #)” adds the two numbers together.
    - Ex. (1 + 1) = 2
- *Subtraction*
  - “(# - #)” subtracts the two numbers.
    - Ex. (3 - 1) = 2
- *Multiplication*
  - “(# * #)” multiplies the two numbers.
    - Ex. (3 * 2) = 6
- *Division*
  - “(# / #”) divides the two numbers.
    - Ex. (6 / 2) = 3
- *Modulo*
  - “(# % #)” takes the modulo of the two numbers (i.e., finds remainder).
    - Ex. (6 / 2) = 0 (divides evenly)
- *Power*
  - “(# ^ #)” takes the first number to the power of the second.
    - Ex. (3 ^ 2) = 9
- *Distance*
  - “(# to #)” finds the distance between the two nodes (can be place ids, such as 0 and 5, or raw ids, such as 10000 and 10005).
    - Ex. (3 to 0) is the distance from node place 3 to the default node place 0
    - Ex. ((TEMP 0) to 0) is the distance from temp node 0 to default node place 0
- *Trigonometry*
  - “(TAN #)” returns the tangent of the number, where the number is taken to be in radians.
    - Ex. (TAN 0) = 0
  - “(SIN #)” returns the sine of the number, where the number is taken to be in radians.
    - Ex. (SIN 0) = 0
  - “(COS 0)” returns the cosine of the number, where the number is taken to be in radians.
    - Ex. (COS 0) = 1
- *Integerization*
  - “(FLOOR #)” returns the number rounded down.
    - Ex. (FLOOR 1.6) = 1
  - “(CEIL #)” returns the number rounded up.
    - Ex. (CEIL 1.6) = 2
  - “(ROUND #)” returns the number rounded.
    - Ex. (ROUND 1.6) =2

- *Randomization*
    - “(RANDOM #)” returns a random number between 0 and the specified number.
        - Ex. (RANDOM 2) = 0 or 0.1 or 1 or 1.5 or 2 or 1.975, etc.
- *ID*
    - “(ID #)” takes in an ID, such as a raw ID, place ID, or combined-layer ID, and reduces it.
        - Ex. (ID 10005) = 5 (a raw ID)
        - Ex. (ID 20003) = 3 (a combined-layer ID)
        - Ex. (ID 15) = 15 (a place ID)
    - “(NODE #)” takes in a raw ID and reduces it.
        - Ex. (NODE 10005) = 5
    - “(COMBINE #)” takes in a combined-layer ID and reduces it.
        - Ex. (COMBINE 20003) = 3
- *Header*
    - “(TEMP #)” returns the raw ID of the node associated with the specified temporary marker.
        - Ex. (TEMP 3) = 10015 if node 15 has “$TEMP 3”
    - “(BACK #)” returns the raw ID of the node that is the specified number of spaces backwards in the previous node list.
        - Ex. (BACK 1) gets the previous node
        - Ex. (BACK 2) gets the node before that
        - Ex. (BACK 0) gives the current node
- *Functions*
    - “(VAR #)” returns the value of the specified variable.
        - Ex. (VAR 1) returns the value of variable 1
- *Fetch*
    - “(# . ?)” returns the value of a property of the specified node. If you enter a place ID or raw ID, it will return the value of a node. If you enter a combined-layer ID, it will return the value of a combined-layer node. The names of the acceptable properties are listed below.
        - Valid Node Properties:
            - COLOR_RED
            - COLOR_GREEN
            - COLOR_BLUE
            - CIRCLE (1 if it’s a circle, 0 otherwise)
            - CIRCLE_RADIUS
            - RADIUS (see above)
            - CIRCLE_INNER_RADIUS
            - INNER_RADIUS (see above)
            - PRECISE (1 if it’s precise, 0 otherwise)

- RECT (1 if it's a rectangle, 0 otherwise)
- RECT_LEN
- RECT_LENGTH
- RECT_WID
- RECT_WIDTH
- RECT_START (returns 0)
- PERPSPACE
- CORNER (1 if it's a corner, 0 otherwise)
- CORNER_BASE
- CORNER_HEIGHT
- CORNER_ANGLE
- CORNER_CURVE
- POLY (1 if it's a polygon, 0 otherwise)
- POLYGON (see above)
- POLY_LEN
- POLYGON_LEN (see above)
- POLY_WID
- POLYGON_WID (see above)
- POLY_VS (returns number of vertices)
- POLYGON_VS (see above)
- POLY_VERTS (see above)
- POLYGON_VERTS (see above)
- POLY_VERTICES (see above)
- POLYGON_VERTICES (see above)
- BUFFER_X
- BUFFER_LEN (see above)
- BUFFER_Y
- BUFFER_WID (see above)
- EXTRA_X
- EXTRA_LEN (see above)
- EXTRA_Y
- EXTRA_WID (see above)
- LAYER
- Z (see above)
- CUT (1 if it will be cut, 0 if not)
- NEIGHBORS (number of attached nodes)
- ANGLE
- STARTANGLE (see above)
- SANGLE (see above)
- NEIGHBOR(?)_#

- Returns the value of the ? property of neighbor #
- Ex. NEIGHBOR(ANGLE)_0 returns the angle of the first neighbor (which is always the node that spawned this one)
- Ex. NEIGHBOR(NEIGHBOR(POLY)_0)_0 returns whether the parent node of this node's parent node is a polygon

- NEIGHBOR_# (returns the raw ID or place ID of the node that is the #th neighbor of the current node)
    - Ex. NEIGHBOR_0 returns the raw id of the parent node of this node
- NEIGHBOR_ANGLE_# (returns the angle of the #th neighbor of this node
    - Ex. NEIGHBOR_ANGLE_0 returns the angle of this node's parent node
- NEIGHBOR_LAYER_# (returns the layer of the #th neighbor of this node)
    - Ex. NEIGHBOR_LAYER_0 returns the layer of this node's parent node
- NEIGHBOR_Z_# (see above)
- NEIGHBOR_DIST_# (returns the distance of this node from its #th neighbor.
    - Ex. NEIGHBOR_DIST_0 returns the distance of this node from its parent node.

▪ Valid Combined-Layer Node Properties:
- SOURCE (id of the combined-layer node that is the parent of this node)
- DIR (direction of this node from its parent, 0 = down, 1 = left, 2 = up, 3 = right)
- ROTATE (rotation of this node, 0 = 0, 1 = 90, 2 = 180, 3 = 270)
- LAYER (which layer this node represents)

References

References are snippets of code that can be replicated on demand elsewhere in a script. First, a reference is defined (this should occur before it is used, it is usually best practice to include references at the top of each script prior to any other code), and then it is called upon later in the code. References do not have to be defined within the script-- references can be saved in the "references" folder and called from there.

- *Establishing References*
    - “{ name” (without a closing bracket) marks the start of a reference. At the end of the reference definition, “}” must be placed alone on its own line.
        - Ex. “{ testref” “>ANGLE 30” “$RECT 4 2” “$BUFFER 1 1” “}” defines a new reference that makes a rectangle node at a 30 degree angle from the current node.
- *Calling References*
    - “{ name }” calls back a reference with the specified name or number to be replicated here. This command can be followed by “x” and a number to repeat this command that number of times. This command can also be followed by “ANGLE” and a number to add that number to all of the angles in this reference. Finally, this command can also be then followed by up to 9 numbers to set variables 0-8 to those numbers.
        - Ex. “{ 11 } x 3” calls back reference 11 and repeats it 3 times
        - Ex. “{ quicktriangle } ANGLE 10” calls back reference “quicktriangle” and moves all of its angles over 10 degrees.
        - Ex. “{ 8 } x 2 ANGLE 30” calls back reference 8, repeats it twice, and moves all of its angles by 30 degrees.
        - Ex. “{ 0 } x 3 ANGLE 15 VARS 1 2.5 4” calls back reference 0, repeats it 3 times, adjusts its angles by 15 degrees, and sets variable 0 = 1, variable 1 = 2.5, and variable 2 = 4
        - Ex. “{ testref } VARS 3 9 55.5” calls back reference “testref” and sets variable 0 = 3, variable 1 = 9, and variable 2 = 55.5.
        - Note: when called, the code from the reference is essentially copied and pasted into the position where the “{ }” call is.

Combine Layer Commands

Combined-layers are used for making arrays of layers. They should be constructed at the end of the script, after the “$FILL” command has been called. Combined-layers are defined by using the “#COMBINE” command followed by a number, which creates a new combine map with the specified number as its ID. Each combined-layer must also have its own “#FILL” command at its end. Unlike the regular “$FILL” command, however, the “#FILL” command does not take a scaling value and only takes a filename.

- *Combine*
    - “#COMBINE” and a number defines a new combined-layer
        - Ex. “#COMBINE 1” makes a new combined-layer, of ID 1

- *Combined-layer nodes*
  - "#ID" and a number defines a new combined-layer node, of the specified ID. Each combined-layer node is a copy of an existing layer, placed in the grid.
    - Ex. "#ID 0" defines a new node of ID 0
  - "#LAYER" and a number specifies which layer a node represents.
    - Ex. "#LAYER 1" states that the current combined-layer node represents layer 1.
  - "#UP", "#LEFT", "#DOWN", or "#RIGHT" and a number determine which node the current combined-layer node is attached to, and which direction from that parent node this new node is.
    - Ex. "LEFT 0" places the current node to the left of ID node 0.
- *Modification*
  - "#ROTATE" and 0, 90, 180, or 270 specifies the rotation of this combined-layer node. The layer image will be rotated by this factor to draw this node.
    - Ex. "ROTATE 180" means this layer will be rotated 180 degrees
  - "#FLIPX" states that this layer will be flipped horizontally when drawn.
  - "#FLIPY" states that this layer will be flipped vertically when drawn.
- *Function*
  - "#TEMP" and a number define a temporary marker for this combined-layer.
    - Ex. "#TEMP 1" sets this combined-layer to be associated with temporary marker 1.
    - Note: the value parser will intuit whether combined-layer temporaries or standard node temporaries are being referenced by the "(TEMP #)" operator automatically.
  - "#VAR" and two numbers defines the value of a variable, as it would for "$VAR".
    - Ex. "#VAR 0 15.3" sets variable 0 equal to 15.3
- *Global*
  - "#FILL" and some text defines the filename of this combined-layer, and indicates the end of this combined-layer.
    - Ex. "#FILL comb_1"
  - "#DRAWBOXES" or "#BOX" indicates that lines should be drawn between each layer in the array.
  - "#SQUARESPACE" or "#SQUARE" indicates that each layer in the array will be put in a grid where each cell is of equal length and width, rather than varying based on layer size.
  - "#CENTER" and a number defines the center point of this combined layer, where the number is the id of a node. The position of that node will be considered the center, and no matter how each layer is rotated or flipped, that node will always be in the center of each cell.
    - Note: this function requires "#SQUARESPACE" to be enabled.

Printing Sizes

There are two methods that can be utilized to print devices. The first—and recommended route—is simply to use the PDF files that are automatically generated by the interpreter, as any printer will recognize these files and print them to scale. In order to scale a device for PDF printing, the proper $FILL conversion must be applied. A $FILL conversion of 11.81 corresponds to a 1-mm scale (e.g., a rectangle of length 2 and width 1 is now a 2 mm × 1 mm rectangle), while a $FILL conversion of 300 corresponds to a 1-inch scale (e.g., the aforementioned rectangle is a 2 in × 1 in rectangle). AutoPAD exports to PDFs at a resolution of 300 pixels per inch, which is 11.81 pixels per mm.

To resize the PDF files that are provided, the $PDF_SIZE_IN and $PDF_SIZE_CM commands are provided. For instance, to generate A6-sized (10.5 cm × 14.8 cm or 4.13 × 5.83 in), "$PDF_SIZE_CM 10.5 14.8" or "$PDF_SIZE_IN 4.13 5.83" would be used. Additionally, by default a half-inch margin is kept on each side of the PDF page. In order to adjust the margin, the $PDF_MARGIN_IN and $PDF_MARGIN_CM commands are provided. For instance, to reduce the horizontal margins to a quarter inch and expand the vertical margins to an inch, "$PDF_MARGIN_IN 0.25 1" would be used. All of these commands are summarized in the global node properties section of this Glossary.

The alternative route is to create printable files out of the images produced manually. There are many ways to do so, but in some cases the fill conversion that corresponds to the pixels-per-inch of the printer must be used. PPI or pixels-per-inch is the size conversion at which a document is printed. For instance, if a 1000 by 1000 pixel image was printed at 200 PPI, the paper would need to be 5 by 5 inches. Hence, if an image is generated with a $FILL conversion of 200, printing that image at 200 PPI means that the lengths used in the script are now inches (ex. a circle of radius '1' is now 1 inch instead of an arbitrary length). The following table provides a look at common paper sizes and their corresponding pixel sizes for three common PPI values. For instance, if printing on Letter paper was desired, and the printer was set to 300 PPI, the image could be a maximum of 2550 × 3300 pixels, and the $FILL conversion should be 300. It is possible to print a smaller than maximum size image without issue, so long as the PPI is the same.
