# Supplementary material for: An Open Software Platform for the Automated Design of Paper-Based Microfluidic Devices
Source: Sci Rep. 2017 Nov 24;7:16224. doi: 10.1038/s41598-017-16542-8 (PMC5701164; doi:10.1038/s41598-017-16542-8)
Supplement: Supplementary file 3 — Introduction to the Tree Interface [file 41598_2017_16542_MOESM3_ESM.pdf]

An Open Software Platform for the Automated Design of Paper-Based Microfluidic Devices

Nicholas S. DeChiara, Daniel J. Wilson, and Charles R. Mace*

Department of Chemistry, Tufts University, 62 Talbot Avenue, Medford, MA 02155 USA

*Corresponding author: charles.mace@tufts.edu

Supplementary Information 3: Introduction to the Tree Interface (9 pp)

The Tree Interface

The **Tree Interface** may be used to recreate nearly any possible script, usually with considerably less effort. However, it is necessary to read the first few sections of the **Getting Started** guide (**Supplementary Information Document 2**) in order to become familiar with the structure of the AutoPAD software. In general, we advise any user to reference the **Getting Started** guide prior to using a specific functionality of the Interface—that is, this guide itself will inform the user of how to use the **Tree Interface**, but the **Getting Started** guide will inform the user of how this usage comes together into a coherent design.

To summarize the various parts of the **Tree Interface**:

**Tree Interface Components**	
**Left-Hand Panel or Editing Panel**	This panel contains all of the buttons and text boxes needed to add nodes and add data to those nodes.
• Header Node Panel	Contains all buttons for adding, clearing and modifying header nodes.
• Rect Tab	Contains all commands related to rectangles.
• Circ Tab	Contains all commands related to circles
• Poly Tab & II Tab	Collectively contain all the commands related to polygons.
• Reference Tab	Contains all commands related to creating reference callbacks. See References Tab for defining references.
• Properties Tab & II Tab	Contains various commands for setting the properties of nodes, such as color, spacing, cut preferences and so on.
• Global Tab	Contains commands for global properties such as filling and buffer boxes.
• PDF Tab	Contains commands for specifying PDF output settings, such as page size.
**Node-Tree Panel**	Contains the tree that displays all node data.<br>Selecting a node makes that header node the current node to edit, so added properties will be set to that node.

**References Panel**	Contains the tree that shows all reference definitions.<br>Selecting reference definitions and header nodes in this panel selects them for editing, so new properties will be added to that node.
• New Reference Panel	Contains the means for adding a new reference definition.
**Combine Panel**	Contains the tree for defining combined-layers.<br>Selecting Combines or IDs sets that node to be the current node, and new combined-layer properties will be applied to that node.
• Combine Properties Panel	Contains all the buttons needed for defining combined-layer properties, adding new IDs and adding new combine definitions.
**Menu Bar**	Contains various menus.
• File Menu	Contains buttons for saving and opening script files.
• Edit Menu	Contains buttons for clearing and editing data.
• Build Menu	Contains buttons for targeting an output directory and for running a script through the interpreter.
• View Menu	Contains buttons for altering the view of the Interface.

Using the Tree Interface requires the application of a few simple principles:

Tree Interface Principles

1. There are two types of nodes: Header nodes and Property nodes.
    a. Header nodes: “>” commands, for instance, can have child nodes.
    b. Property nodes: “$” commands, for instance, cannot have child nodes.
2. Only Header nodes can be selected.
    a. Selecting a Property node simply selects the Header node that is its parent.
3. Only one node can be selected at a time.
4. New nodes are added as children of the currently selected node.
5. In general, trying to add a Property node that is a duplicate of an existing node removes the existing node instead.
6. When a new Header node is created, it becomes selected.

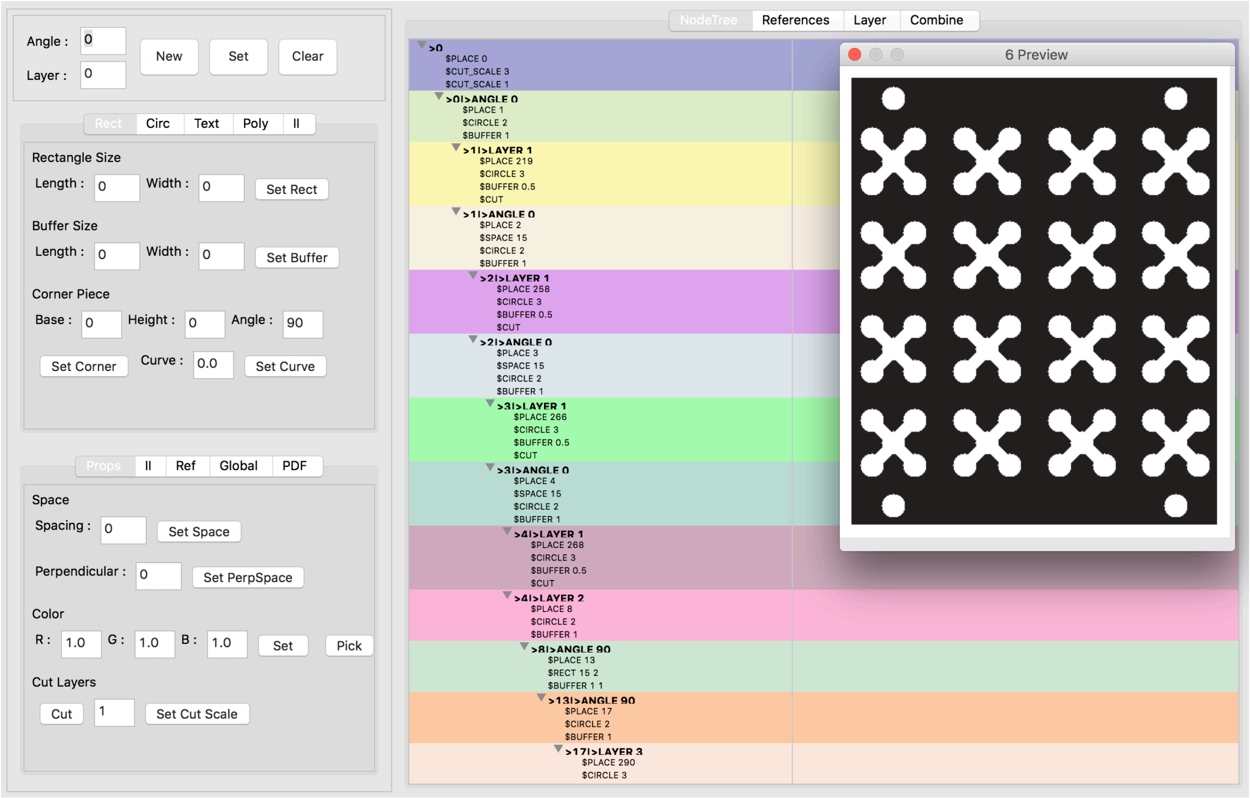

The AutoPAD Tree Interface

Each design begins with the “>0” Header node. This node is not removable, and represents the “origin node” from which the rest of the design must branch. Additionally, the “>0” node always has the “$FILL” Property node attached to it, which is not removable. All elements of each design are placed in-between these two bookends, such as all of the other nodes.

Pattern recognition will be of considerable use in designing going forward, so consider the following ways that various structures may be represented:

Visual	Verbal	Script	Node-Tree
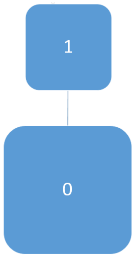	One node attached to another node.	>0<br>>UP	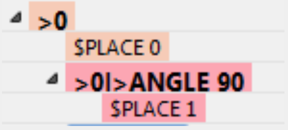
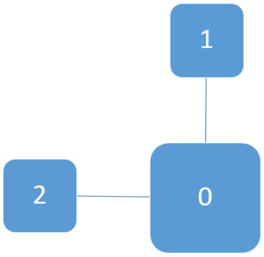	Two nodes attached to a single node.	>0<br>>UP<br>>0<br>>LEFT	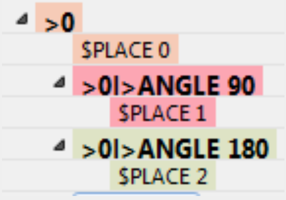
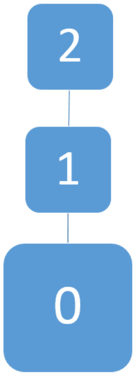	Three nodes in a chain.	>0<br>>UP<br>>UP	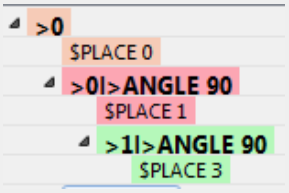
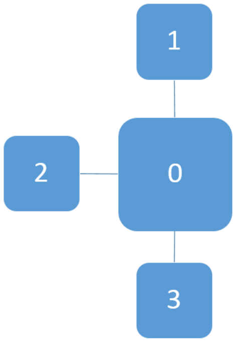	Three nodes attached to a single node.	>0<br>>UP<br>>0<br>>LEFT<br>>0<br>>DOWN	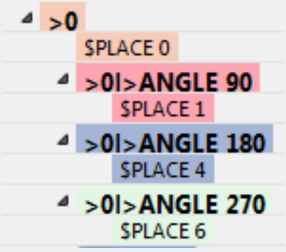

Recognizing structures in each of these forms will greatly accelerate the process of reading and understanding designs. Note as well that the tabbing in the node-tree lines up such that each child node is one tab forward from the node that parented it, and also note that this is achieved by collapsing the “>#” command into each header node (most visibly in the last example). This formatting is expanded when the file is saved, but until then this compression offers viewing clarity. Additionally, in the node-tree every header node is automatically given a place ID. This is to allow the “>#” compression to function.

Adding header nodes is straightforward. The top-left panel is the new node panel, use the **New** button to add a new node, the **Set** button to change the currently selected node, and the **Clear** button to remove the currently selected node (and all of its children). If the layer in the textbox is the same as the currently selected node, the new node will be an “>ANGLE” node. If the layer is different, however, the new node will be a “>LAYER” node. After making a new header node, that new node becomes the currently selected node automatically. When building layers, it is sometimes useful to access the Layer tab and select a layer to view. The **Layer** tab will display only a single layer at a time, which can clear up confusion in the design. Additionally, ‘Color-by-Layer’ mode is automatically enabled. This mode colors nodes by their layer, so nodes on the same layer will have the same background color. This can be toggled at any time via the **View** menu, and the colors may be swapped with new, random colors by selecting the **Change Colors** button from the same menu.

Once nodes are in place, they can be selected via mouse-click and given properties. Navigating to the **‘circ’** tab allows for a node to be set as a circle, the **‘rect’** tab allows for rectangles, and so on. Operation of all of these panels is straightforward: select the node to edit, fill the relevant text boxes, and press the relevant button. Most designs may be recreated simply with the rectangle and circle settings:

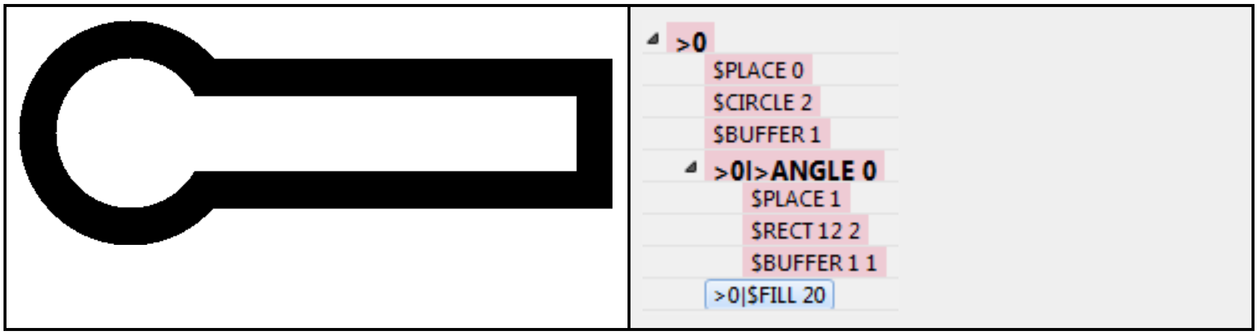

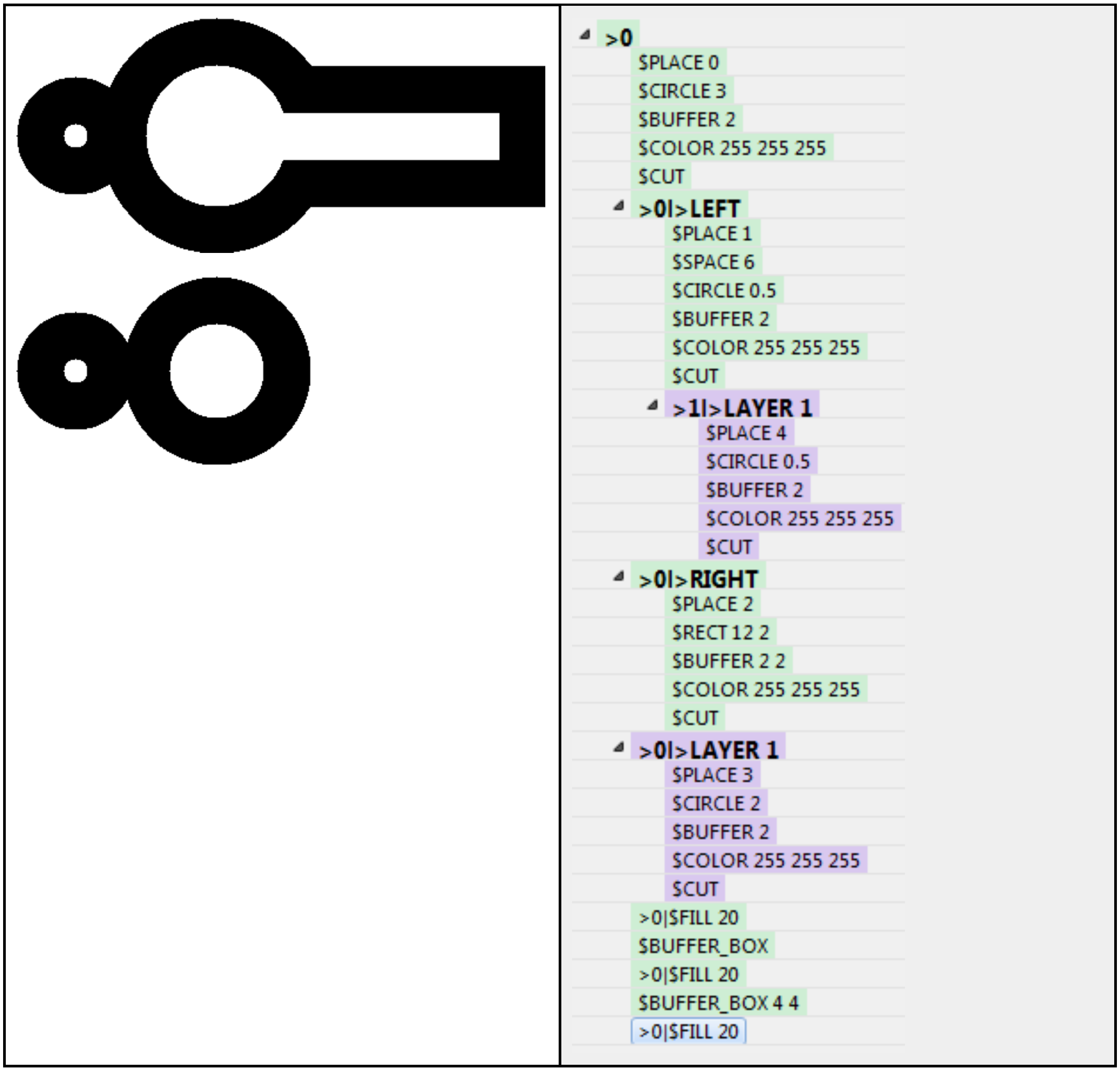
>0
$PLACE 0
$CIRCLE 3
$BUFFER 2
$COLOR 255 255 255
$CUT
>0|>LEFT
$PLACE 1
$SPACE 6
$CIRCLE 0.5
$BUFFER 2
$COLOR 255 255 255
$CUT
>1|>LAYER 1
$PLACE 4
$CIRCLE 0.5
$BUFFER 2
$COLOR 255 255 255
$CUT
>0|>RIGHT
$PLACE 2
$RECT 12 2
$BUFFER 2 2
$COLOR 255 255 255
$CUT
>0|>LAYER 1
$PLACE 3
$CIRCLE 2
$BUFFER 2
$COLOR 255 255 255
$CUT
>0|$FILL 20
$BUFFER_BOX
>0|$FILL 20
$BUFFER_BOX 4 4
>0|$FILL 20

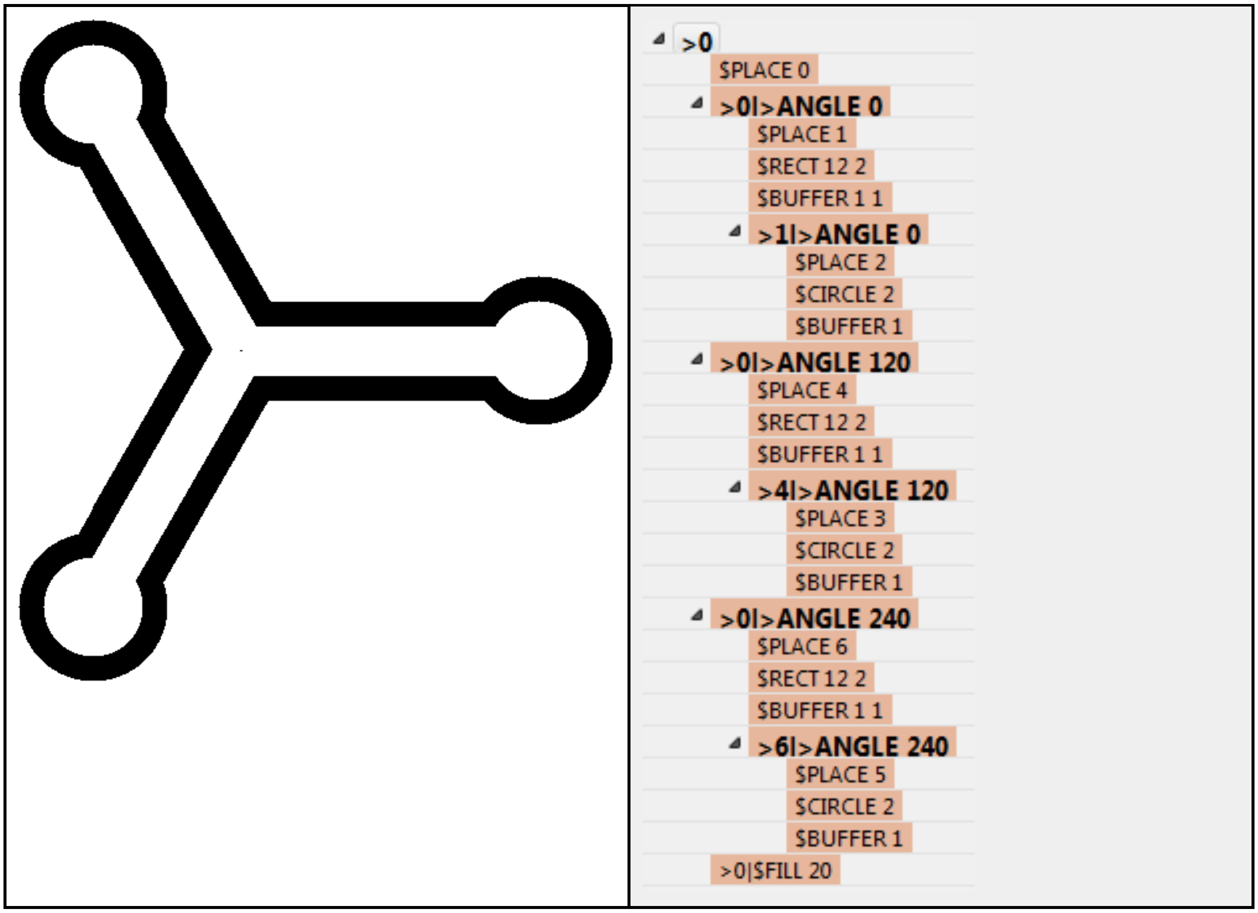

Once a design is finished, navigate to the **File** menu and **Save** the current script. Then, the script may be run directly from the **Build** menu. If a specific output location is desired, select a **Target** from the **Build** menu (otherwise, it will default to the /out/ folder). To start on a fresh design, the **Clear** command may be accessed from the **Edit** menu.

The features of references and combined-layers may also be accessed from the Tree Interface. In the **References** tab adjacent to the **NodeTree** tab, use the **Create** button to make a new reference of the specified title. To remove a reference, use the **Create** button on its title a second time. References may be filled with header and property nodes in the same way that the regular node-tree nodes may be: simply select part of the reference and start using the same buttons and panels as would be used in the node-tree. To use a reference in the node-tree, use the **Reference** tab in the shape-panel. Specify the title and angle adjustment, select the position to add the reference callback, and press “Add”. The references added here will automatically be appended to the start of the script file when saving.

To use combined-layers, access the **Combine** tab in the top-right. Create a new combine of a given ID, and then it may be filled with various layers. To add a layer, use the panel beneath the **Create Combine** panel. Specify an ID and a layer it will be based on, then press **New ID**. If this is not the first layer, it must have a source specified and a direction to come from, which can be specified by pressing the **Direction** button. Then, apply flipping or rotation as is desired. The

combined-layers created here will automatically be appended to the end of the script file after saving.

In addition to compiling images of each layer, the Tree Interface will create a PDF per layer as well. By default, this PDF is in the standard US letter size, and the device is duplicated as many times in each direction as will fit the page, subject to the margin sizes (which are half-inch, by default). The PDF tab contains options for changing these settings, as the page and margin sizes may be specified to whatever values are desired.
