# Supplementary material for: An Open Software Platform for the Automated Design of Paper-Based Microfluidic Devices
Source: Sci Rep. 2017 Nov 24;7:16224. doi: 10.1038/s41598-017-16542-8 (PMC5701164; doi:10.1038/s41598-017-16542-8)
Supplement: Supplementary file 4 — Getting Started with AutoPAD [file 41598_2017_16542_MOESM4_ESM.pdf]

# An Open Software Platform for the Automated Design of Paper-Based Microfluidic Devices

Nicholas S. DeChiara, Daniel J. Wilson, and Charles R. Mace*

Department of Chemistry, Tufts University, 62 Talbot Avenue, Medford, MA 02155 USA

*Corresponding author: charles.mace@tufts.edu

**Supplementary Information 4: Getting Started with AutoPAD (41 pp)**

## Table of Contents


| | | |
|---|---|---|
| **1.** | **Conceptualizing** | pg 2 |
| **2.** | **Tools** | pg 2 |
| **3.** | **Creating** | pg 4 |
| **4.** | **Node Commands** | pg 5 |
| **5.** | **Property Commands** | pg 7 |
| **6.** | **Fill Command** | pg 8 |
| **7.** | **Basic Shapes & Precision** | pg 10 |
| **8.** | **Corners and Curves** | pg 12 |
| **9.** | **Text** | pg 14 |
| **10.** | **Layers** | pg 15 |
| **11.** | **Cut Layers** | pg 18 |
| **12.** | **Buffer Boxing** | pg 20 |
| **13.** | **Polygons** | pg 21 |
| **14.** | **Temp and Back** | pg 25 |
| **15.** | **Operations & Variables** | pg 27 |
| **16.** | **References** | pg 29 |
| **17.** | **Combined-Layers** | pg 35 |
| **18.** | **Printing and PDFs** | pg 41 |

## Conceptualizing

Within the AutoPAD device drawing system, all information is carried by "*nodes*". A node is a block of data that has a position relative to other nodes. For example in **Figure 1**, nodes 1, 3 and 4 are placed relative to node 0. If node 0 is moved, these three nodes will follow. Similarly, node 2 is attached to node 1 and will follow it. If node 0 moves, it will take node 1 with it, which will in turn move node 2 as well. As a result of this system, an individual node only needs to care about which node it is attached to, and thus the data that each node carries is completely localized and can be modified without needing to modify other nodes. The data that these nodes carry inform what the node will look like when it is rendered by the system. This is exemplified by **Figure 2**: each node carries with it all the necessary information to draw a shape and define its position, and the system has support for all simple polygons and many types of curved shapes. Taken together, these nodes use their individual shapes to form a full image.

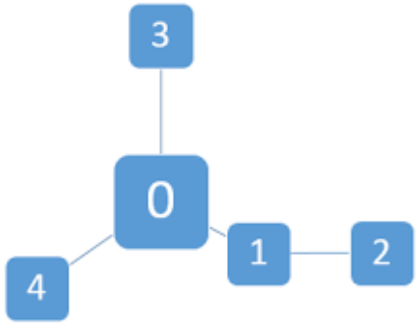


**Figure 1.** Relative positions of nodes.

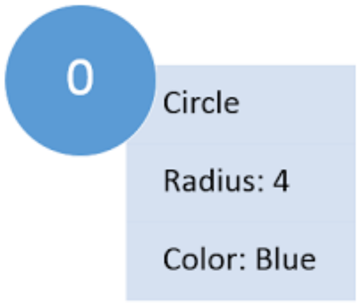


**Figure 2.** Information within a node.

## Tools

In order to store and operate on node data, the AutoPAD device drawing system utilizes an extremely basic scripting language. Data elements are stored in text files written with this language, and the programs in the AutoPAD suite are designed to parse and work upon data stored in this manner. As a result, patterns can be created directly from text files using the build interface (**Figure 3**). AutoPAD uses a program called Interpreter to construct images out of node data. The Interpreter loads text files written in the AutoPAD scripting language and parses the data therein, finally outputting the images described by that node data. The Interpreter is central; everything else within the suite is designed around interfacing with the Interpreter, and around building scripts for its use.

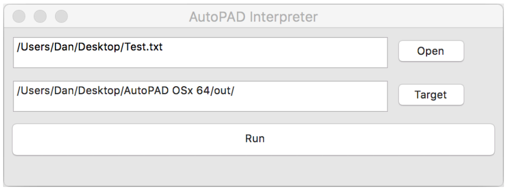


**Figure 3.** Loading text files.

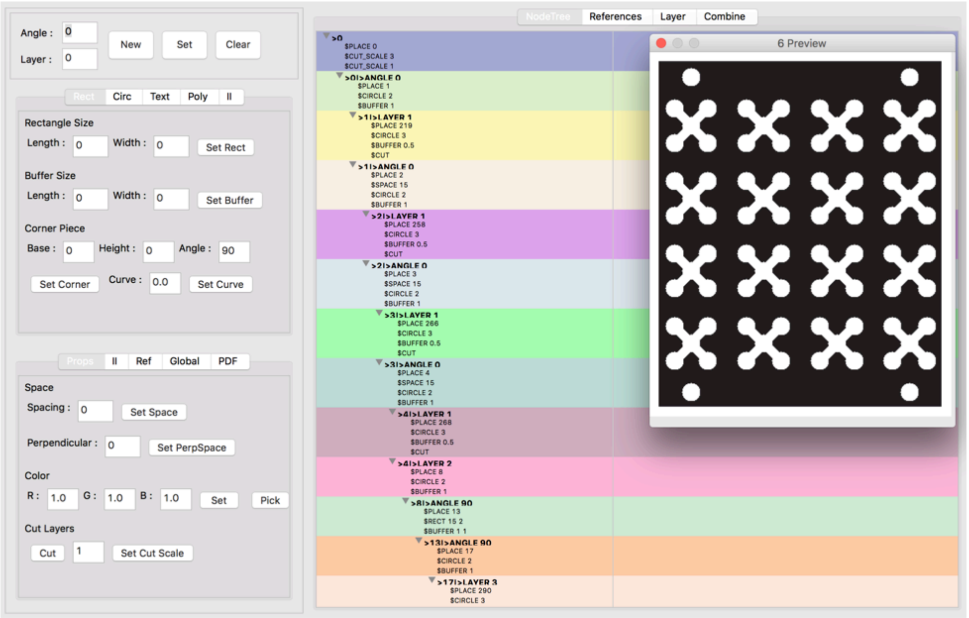


**Figure 4.** Screen shot of AutoPAD device drawing interface.

## Creating

Learning to understand and write in the AutoPAD scripting language is simple, especially for users with coding experience. Though there are many commands within the language, the syntax and structure are simple. As an important note, it is possible to utilize the system entirely through the Tree Interface and it is not necessary to memorize any or all of the information that follows about the structure of the language. Hence, the structure of most designs is simple: each '>' command specifies a new node, and is followed by several '$' commands that specify properties of that node. Some designs will choose to use some of the advanced commands, but these are unnecessary and provided only to speed up development for experienced users. Nevertheless, these syntax rules apply to those commands as well.

**Keystones of Syntax**

1. Each command has its own line (i.e. two commands cannot be on one line).
2. Commands start with a defining character that indicates its type.
   a. > indicates a Node (or a Header) command, signifying a new node.
   b. $ indicates a Property command, specifying some data in the current node.
   c. #, { and } symbols are used for some advanced features which will be discussed later.
   d. " indicates a comment, which means this line won't be read.
3. Input parameters are separated by spaces (compared to, say, commas of other languages).
4. Operations are indicated by use of parentheses. Each operation must be enclosed within its own set of parentheses. For example:
   a. (1 + 3) = 4
   b. (2 * (4 - 1)) = 6
5. Case does not matter.

## Node Commands

As described above, each script begins with the application of ‘>’ commands. The goal of scripting is essentially to create nodes and fill them with data, and thus nothing can be designed prior to the creation of nodes. There are several ‘>’ commands, but these are the necessities. Then, these nodes are filled with data by the ‘$’ commands. Each ‘$’ command refers to the current node, which means the most recent ‘>’ command. It is important to note that having more than one branch off of a single node requires the use of the $PLACE command for this same reason: once the first branch is created, adding another node would be adding onto that branch. Instead, the script must go back to the original node, and to do that places must be used.

| **Basic ‘>’ Commands** | |
|---|---|
| **>ANGLE #** | Where # is an angle in degrees, used to create a new node at a given angle from the current node (e.g. >Angle 30 creates a node at the 30 degree mark). |
| **>LAYER #** | Where # is a layer number, used to create a new node on a given layer, at the position of the current node (e.g. >Layer 2 creates a new node on the second layer). |
| ># | Where # is a number is used to return to a specific, already existing node (e.g. >0 returns to the origin node). |
| *Refer to the Glossary for a full list of ‘>’ commands.* | |

To create the distribution shown in **Figure 1**, for example, the following script would be used (note that quotation marks denote a comment line and not data):

```
>0
        “ Lines starting with quotations are ignored
        “ Node 0 is created by default as the origin node
        >ANGLE 330
                $PLACE 1
                “ Note how node 0 isn’t returned to, giving the 1 -> 2 sequence
                >ANGLE 0
                        $PLACE 2
>0
        “ Go back to node 0
        >ANGLE 90
                $PLACE 3
>0
        >ANGLE 225
                $PLACE 4
```

That is, in order to create multiple branches off of node 0, it must be returned to each time, because adding a new node makes that new node the current node. Also, note the convention of tabbing lines; similar to a bullet-point list, commands that are tabbed over further over are within the command one level up. Consider the same script formatted via bullet point:

- >0
    - “ Lines starting with quotations are ignored
    - “ Node 0 is created by default as the origin node
    - >ANGLE 330
        - $PLACE 1
        - “ Note how node 0 isn’t returned to, giving the 1 -> 2 sequence
        - >ANGLE 0
            - $PLACE 2
- >0
    - “ Go back to node 0
    - >ANGLE 90
        - $PLACE 3
- >0
    - >ANGLE 225
        - $PLACE 4

Note, however, that tabbing is unnecessary, and the user can ultimately use any convention without incident. The Interpreter ignores all spacing before and after commands, so spacing is for the viewer’s convenience alone. The cardinal directions can be accessed via the shortcut commands, >LEFT, >RIGHT, >UP and >DOWN. Consider the following example:

| | |
|---|---|
| >0<br>>RIGHT<br>$PLACE 1<br>>UP<br>$PLACE 2 | >0<br>>RIGHT<br>$PLACE 1<br>>0<br>>UP<br>$PLACE 2 |
| 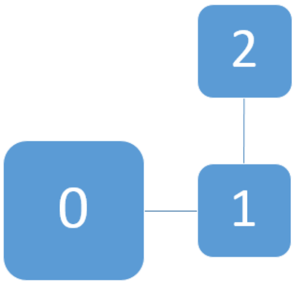 | 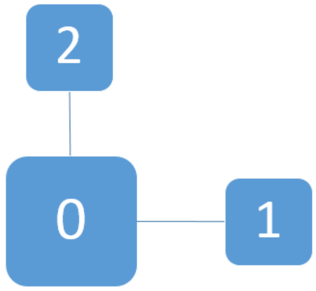 |

## Property Commands

Filling these nodes with data requires the use of property commands, a few of which are detailed below. Note that commands are identified by the start-of-line symbol and the first word (i.e., everything before the first space). Any other words that are included are ignored. For instance, this means that "$COLOR red 255 green 255 blue 100" is equivalent to "$COLOR 255 255 100", and "$CIRCLE radius 4" is equivalent to "$CIRCLE 4". Ultimately, the user can choose to include as much reminder text as is desired, but the commands below are listed with reminder text so as to alleviate the learning process. The symbol '#' will be used as before to show places where a number should be included.

| **Basic '$' Commands** | |
|---|---|
| **$PLACE id #** | As before, used to mark the node with a specific place ID so that it may be returned to by use of the '>#' command. |
| **$CIRCLE radius #**<br>**$CIRCLE radius # inner #** | Marks the node as a circle of the given radius. If an inner radius is given, the circle will instead be a disk. |
| **$RECT length # width #** | Marks the node as a rectangle of the given length (i.e. in the direction of this node's angle) and width. |
| **$CORNER base # length # angle #** | Marks the node as a corner piece, a triangle of the given base, length and angle. Defines a triangle from two side lengths and the angle between them. |
| **$CORNER_CURVE weight #** | If this node is a corner, gives it a certain amount of curvature-- default is 1.0, and higher values give concavity. |
| **$BUFFER length # width #**<br>**$BUFFER radius #** | Gives the node a black outline border around its shape, either as a box or circle. |
| **$EXTRA length # width #** | Gives the node extra whitespace around its shape. Serves only to push back the image edge. |
| **$SPACE length #** | Gives the node extra space from its parent (or less, if negative). |
| **$PERPSPACE length #** | Gives the node extra space from its parent, laterally. That is, spacing at an angle perpendicular to its source node. |
| **$COLOR red # green # blue #** | Determines the color of the node's shape. Note that these color values are from 0-255. |

| | |
|---|---|
| **$TEXT text** | Marks the node as a text node, displaying the given text. Note that the text will be rotated to the angle of this node. |
| **$TEXT_SIZE size #** | Changes the text scale for this node. Note that this is not the pt. size of the font but the scale of the font relative to the fill size. 1.0 is the default. |
| **$TEXT_FONT fontname** | Changes the font type of this node. |
| **$PRECISE** | Marks this node for precise spacing, which adjusts neighboring nodes so that they touch the edge rather than the center of this node (examples to follow). |
| **$FILL size # filename** | Must be called at the end of every script. Generates a set of images with the given pixel conversion size and filename. |

## Fill Command

First, a note on $FILL: this is one of the few special commands within the language; this command must be called at the end of each script file, as it triggers the actual rendering of the images. Anything coming after the $FILL command will not be included in the image, and additionally it is safer to call this command on the origin node (i.e., by first calling >0). Consider the following examples:

| | | |
|---|---|---|
| >0<br>    >RIGHT<br>        $PLACE 1 | >0<br>    $FILL 1<br>    >RIGHT<br>        $PLACE 1 | >0<br>    >RIGHT<br>        $PLACE 1<br>>0<br>    $FILL 1 |
| No result (the $FILL command was never activated!) | 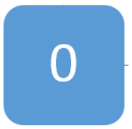  | 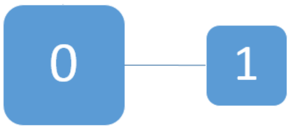  |

The pixel conversion size of the fill command determines how all of the lengths, radii etc. in the script are scaled to pixels. For instance:

| Comparison of $CIRCLE radius 1 | |
|---|---|
| $FILL 1 |  |
| $FILL 10 | 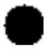 |
| $FILL 100 | 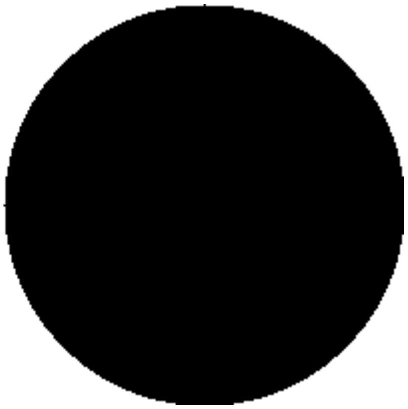 |

A scale of “$FILL 1” means that the lengths in the script are pixel-lengths, so a radius of 10 is a radius of 10 pixels. A scale of “$FILL 10” means there are 10 pixels per each unit length, so a radius of 10 is a radius of 100 pixels, and so on. So long as the scale is greater than 1, decimal lengths and radii can be used (e.g., a circle of radius 0.5 with “$FILL 10” has a radius of 5 pixels). This pixel conversion should be tied to the PPI (pixels-per-inch) of the printer which will be used. For example, if the printer’s PPI is 300, then using a pixel conversion of 300 means that a circle of radius 1 will have a real radius of 1 inch. By these means, the arbitrary lengths within the scripting language may be converted to real sizes.

As a very critical note: while the AutoPAD software can handle extremely large image sizes, it is often wiser to generate images at a lower size and then scale them up (e.g., using size 100 and then external photo editing software to increase the size 3 times). There will be a threshold, at some point, where the memory used by the program to generate an image exceeds the maximum allotment and generation fails, though this threshold should be far beyond regular use. Images over 5000 x 5000 pixels can be generated without issue on most computers. Meanwhile, the standard U.S. printing paper is 8.5 x 11 inches, meaning that at a high PPI (300 is often considered best quality) the required image size would be 2550 x 3300 pixels—much below the limits of the program. A table is provided in the **Glossary** (**Electronic Supplementary Information Document 2**) to aid in determining which sizes should be used.

## Basic Shapes & Precision

The use of ordinary property commands is simple, by comparison: simply include the commands in the node definitions. Consider the following examples:

<table>
<tr><td>>0<br>$CIRCLE radius 2<br>$COLOR 255 0 0<br>$FILL 20 example1</td><td>>0<br>>ANGLE 330<br>$RECT len 8 wid 2<br>$COLOR 255 0 0<br>$FILL 20 example2</td></tr>
<tr><td>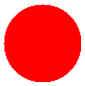</td><td>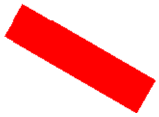</td></tr>
<tr><td>>0<br>$CIRCLE radius 2<br>$COLOR 255 0 0<br>$BUFFER 1<br>$FILL 20 example3</td><td>>0<br>>ANGLE 330<br>$RECT len 8 wid 2<br>$COLOR 255 0 0<br>$BUFFER 1 1<br>$FILL 20 example4</td></tr>
<tr><td>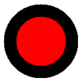</td><td>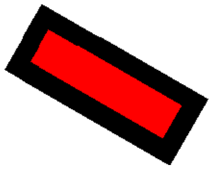</td></tr>
<tr><td>>0<br>$CIRCLE radius 2<br>$COLOR 255 0 0<br>$BUFFER 1<br>>ANGLE 330<br>$RECT len 8 wid 2<br>$COLOR 0 0 255<br>$BUFFER 1 0.5<br>>0<br>$FILL 20 example5</td><td>>0<br>$CIRCLE radius 2<br>$COLOR 255 0 0<br>$BUFFER 1<br>>ANGLE 330<br>$RECT len 8 wid 2<br>$COLOR 0 0 255<br>$BUFFER 1 0.5<br>$PRECISE<br>>0<br>$FILL 20 example6</td></tr>
<tr><td>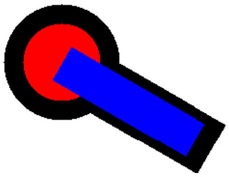</td><td>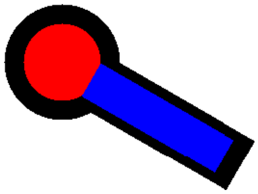</td></tr>
</table>

Most of this is straightforward, but note especially the usage of $PRECISE between the final two examples: without $PRECISE, the above example has its rectangle start directly on top of its parent node. In fact, *all nodes start at the same position as their parent*, until $PRECISE or $SPACE is applied to them. This is seen in the following example where the rectangle is now $PRECISE and is moved to the edge of the circle. $PRECISE functions such that the object in question is placed on a chord within the circle that is the same width as the object, i.e. in this case the rectangle's vertices are on the outer perimeter of the circle. One important caveat—if the object is wider than the circle in question, then $PRECISE will do nothing as no suitable chord exists. $SPACE provides even more control:

```
>0
	$CIRCLE radius 2
	$COLOR 255 0 0
	$BUFFER 1
	>ANGLE 330
		$RECT len 8 wid 2
		$COLOR 0 0 255
		$BUFFER 1 0.5
		$SPACE 6
>0
	$FILL 20 example7
```

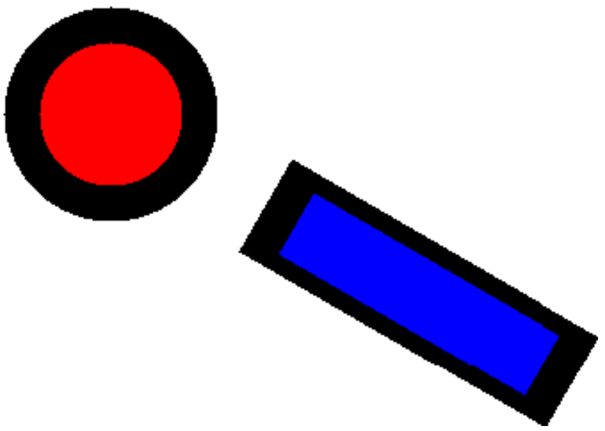

```
>0
	$CIRCLE radius 2
	$COLOR 255 0 0
	$BUFFER 1
	>ANGLE 330
		$RECT len 8 wid 2
		$COLOR 0 0 255
		$BUFFER 1 0.5
		$SPACE -3
>0
	$FILL 20 example8
```

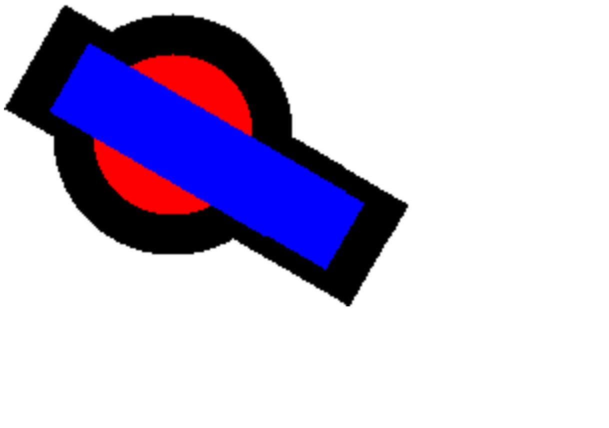

$SPACE can be used to move a node further away from its parent in either direction along the axis of the node's angle (i.e., a positive spacing moves the node further in the direction of its angle, a negative spacing moves the node further in the direction of the opposite angle). $SPACE can be coupled with $PRECISE, and of course the effects of $PRECISE may be replicated with $SPACE, but may require additional calculations.

## Corners and Curves

Corners are used primarily for seamless connection of rectangles that are at differing angles. Corners are specified by two sides (a base and a side) and the angle between them, and form a triangle. Note that any nodes sourced from a corner node will start on the non-base side of this triangle. Also, note that the corner's angle cannot exceed 90 degrees due to engine limitations (for more severe turns, use several corners in a row). By default, the third side of this triangle is simply a straight line that connects the two, but with application of the $CORNER_CURVE command, this third side can be made into a curve instead. Also, note that because triangular buffers would be jagged and inconsistently thick, corner pieces use rectangular buffers. Consider the following examples:

```
>0
	$RECT 4 1
	$BUFFER 0.5 0.5
	>ANGLE 0
		$CORNER 1 1 90
		$BUFFER 0.5 0.5
		>ANGLE 90
			$RECT 4 1
			$BUFFER 0.5 0.5
>0
$FILL 28.35 example37.txt
```

```
>0
	$RECT 4 1
	$BUFFER 0.5 0.5
	>ANGLE 0
		$CORNER 1 1 -90
		$BUFFER 0.5 0.5
		>ANGLE -90
			$RECT 4 1
			$BUFFER 0.5 0.5
>0
$FILL 28.35 example38.txt
```

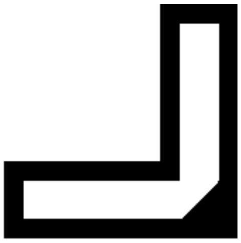

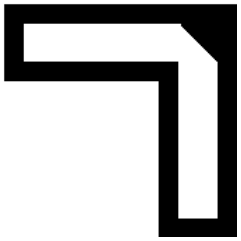

```
>0
	$RECT 4 1
	$BUFFER 0.5 0.5
	>ANGLE 0
		$CORNER_CURVE 1
		$CORNER 1 1 90
		$BUFFER 0.5 0.5
		>ANGLE 90
			$RECT 4 1
			$BUFFER 0.5 0.5
>0
$FILL 28.35 example39.txt
```

```
>0
	$RECT 4 1
	$BUFFER 0.5 0.5
	>ANGLE 0
		$CORNER 1 1 60
		$BUFFER 0.5 0.5
		>ANGLE 60
			$RECT 4 1
			$BUFFER 0.5 0.5
>0
$FILL 28.35 example40.txt
```

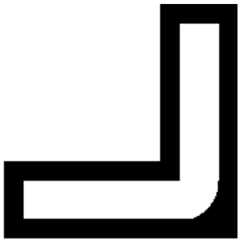

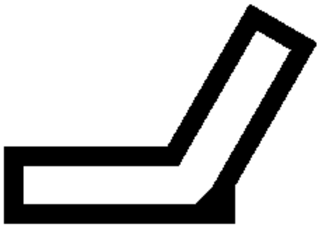

```
>0
 $RECT  4  1
 $BUFFER  0.5  0.5
 >ANGLE  0
  $CORNER  1  1  60
  $BUFFER  0.5  0.5    >ANGLE  60
   $RECT  4  1
   $BUFFER  0.5  0.5
   >ANGLE  60
    $CORNER  1  1  60
    $BUFFER  0.5  0.5
    >ANGLE  120
     $RECT  4  1
     $BUFFER  0.5  0.5
     >ANGLE  120
      $CORNER  1  1  60
      $BUFFER  0.5  0.5
```

*Continued on right.*

```
     >ANGLE  180
      $RECT  4  1
      $BUFFER  0.5  0.5
      >ANGLE  180
       $CORNER  1  1  60
       $BUFFER  0.5  0.5
       >ANGLE  240
        $RECT  4  1
        $BUFFER  0.5  0.5
        >ANGLE  240
         $CORNER  1  1  60
         $BUFFER  0.5  0.5
         >ANGLE  300
          $RECT  4  1
          $BUFFER  0.5  0.5
          >ANGLE  300
           $CORNER  1  1  60
           $BUFFER  0.5  0.5
>0
$FILL  28.35  example41.txt
```

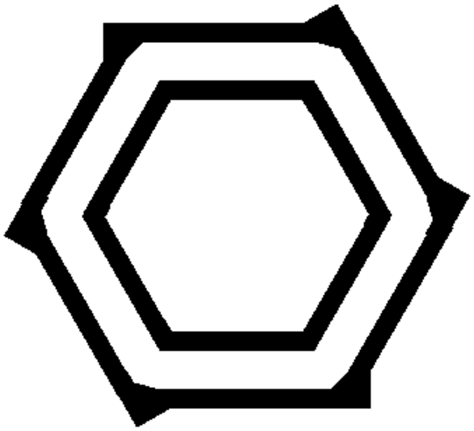

## Text

Text nodes are used primarily for labeling purposes, and simply add text in the color of the node. This is an important note, because the default node color is white, so making text appear usually requires giving the node a specific color. New nodes attached to a text node will appear at the end of the text. Two factors of text nodes may be controlled: font and size. The font can be modified with the $TEXT_FONT command, provided that it is given a font present on the user's system. On computers using OSX operating systems, Font Book may be used to browse for available fonts. The Windows equivalent would be the Character Map utility. Attempts to recreate examples used in this document may fail when they use fonts that are not present on the user's system. The size can be modified with the $TEXT_SIZE command, which describes the ratio of the font's pt. size to the fillsize of the script. The default font size is 1.0, which means that if, for instance, the fillsize were 20, the text would be 20 pt. size, and using a text size of 0.5 would render a 10 pt. font. Consider the following examples:

<table>
<tr>
<td><pre>
>0
	$CIRCLE 1
	$BUFFER 0.5
	>ANGLE 0
		$TEXT Sample Text
		$COLOR 1 0 0
		$SPACE 1.5
>0
	>ANGLE 90
		$TEXT Sample Text
		$COLOR 1 0 0
		$SPACE 1.5
	>0
	$FILL  28.35  example42.txt
</pre></td>
<td><pre>
>0
	$CIRCLE 1
	$BUFFER 0.5
	>ANGLE 0
		$TEXT Sample Text
		$COLOR 1 0 0
		$SPACE 1.5
		>ANGLE 90
			$TEXT Sample Text
			$COLOR 1 0 0
			$SPACE 1.5
	>0
	$FILL  28.35  example43.txt
</pre></td>
</tr>
<tr>
<td>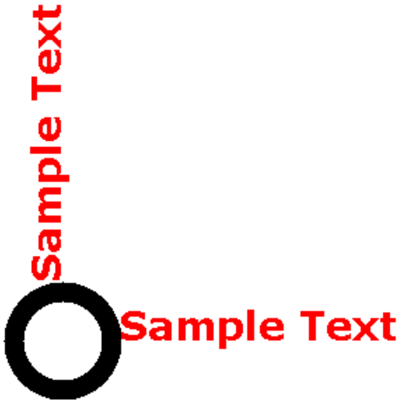
</td>
<td>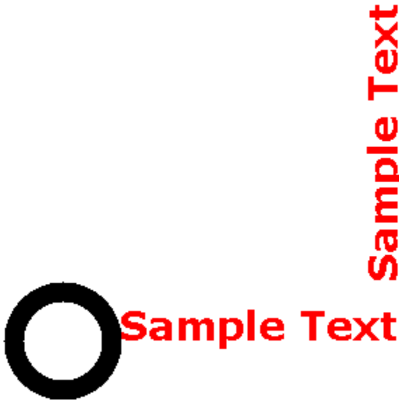
</td>
</tr>
<tr>
<td><pre>
>0
	$CIRCLE 1
	$BUFFER 0.5
</pre></td>
<td><pre>
>0
	$CIRCLE 1
	$BUFFER 0.5
</pre></td>
</tr>
</table>

```
>ANGLE 0
        $TEXT Sample
        $TEXT_SIZE 0.5
        $COLOR 1 0 0
        $SPACE 1.5
        >ANGLE 0
                $TEXT Text
                $TEXT_SIZE 1.5
                $COLOR 1 0 0
>0
$FILL  28.35  example44.txt
```

```
        >ANGLE 0
                $TEXT Sample Text
                $TEXT_FONT MoolBoran
                $COLOR 1 0 0
                $SPACE 1.5
>0
        >ANGLE 0
                $TEXT Sample Text
                $TEXT_FONT Giddyup Std
                $COLOR 1 0 0
                $SPACE 1.5
                $PERPSPACE 1
        >0
        $FILL  28.35  example45.txt
```

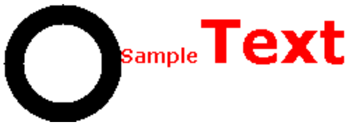


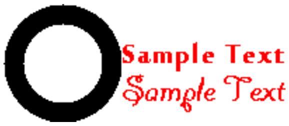


## Layers

Layers are essentially an individual image generated by the same script. New layers are created by making a new node on that layer, by means of the >LAYER command. Layer IDs can be positive or negative, and do not have to be in sequence. Layer IDs should not exceed +/- 2,000,000,000, but otherwise there is no limit to the number of layers present in one script. When a node is made on a new layer, it is automatically aligned with its parent node; that is, if the two layers were overlaid, the new node would lay exactly upon its parent node. Consider the following simple example:

```
>0
        >LEFT
                $PLACE 1
                >LAYER 1
                        $PLACE 2
```

Layer 0

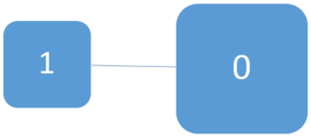


Layer 1

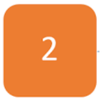


Overlapped Layers*

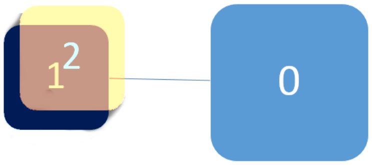


**Skewed for effect*

Thus, this system can easily be applied to align holes between layers. Consider the following example wherein an alignment hole is present in the same position on both layers, and where two wicking zones are overlaid exactly and have different radii:

```
>0
        $CIRCLE 3
        $BUFFER 2
        >LEFT
                " the alignment hole
                $PLACE 1
                $SPACE 6
                $CIRCLE 0.5
                $BUFFER 2
>0
        >RIGHT
                $RECT 12 2
                $BUFFER 2 2
>0
        >LAYER 1
                $CIRCLE 2
                $BUFFER 2
>1
        " return to the alignment hole
        >LAYER 1
                $CIRCLE 0.5
                $BUFFER 2
>0
        $FILL 20 thermo1_layers
```

**Layer 0**

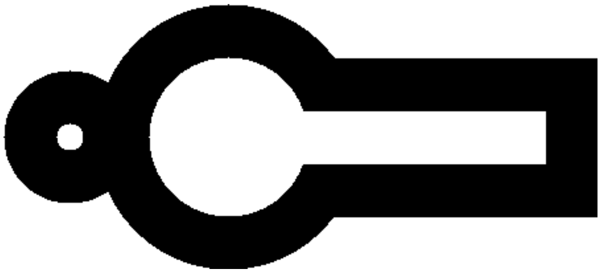

**Layer 1**

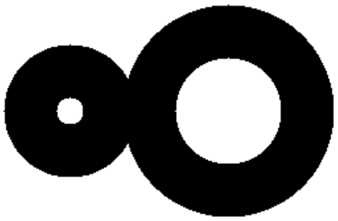

The power of layers should, therefore, be obvious. Alignment between layers is trivial with this system, and additionally the Interpreter will automatically ensure that each layer image is the same size, so that they may be more easily overlaid. Note, also, that nodes on new layers inherit the angle of their parent. The following examples demonstrate this:

```
>0
        >ANGLE 135
                $RECT len 8 wid 2
                $BUFFER 1 0.5
                >LAYER 1
                        $RECT len 8 wid 2
                        $BUFFER 1 0.5
                        >LAYER 0
                                $RECT len 4 wid 2
                                $BUFFER 1 0.5
>0
        $FILL 30 example9
```

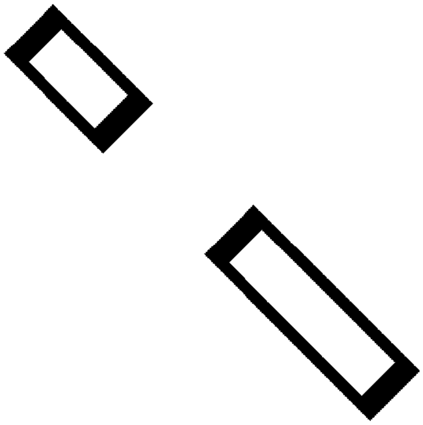

Layer 0

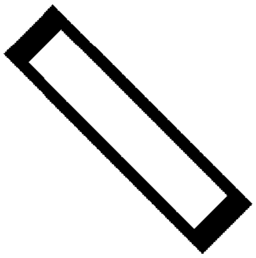

Layer 1

Laid over each other, these two layers would look like the following, if the top layer was translucent:

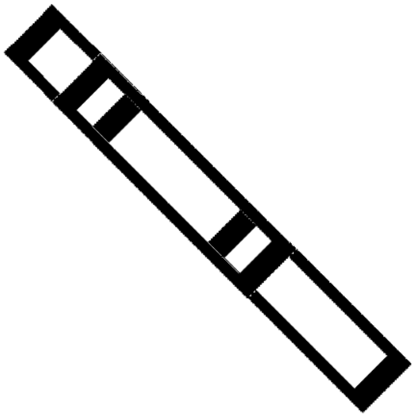

Overlaid

```
>0
    >ANGLE 135
        $RECT len 8 wid 2
        $BUFFER 1 0.5
        >LAYER 1
            >ANGLE 0
                " Changing the angle partway through will be inherited
                $RECT len 8 wid 2
                $BUFFER 1 0.5
                >LAYER 0
                    $RECT len 4 wid 2
                    $BUFFER 1 0.5
>0
    $FILL 30 example10
```

Layer 0

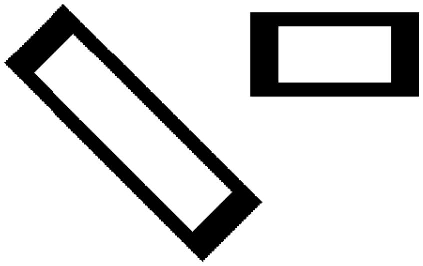

Layer 1

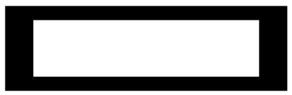

In the first example above, the angle is not changed from the first node, hence even between the layers each following node inherits that angle. In the second example, however, the angle is changed partway through, and it can be seen that this new angle is the one inherited to future nodes.

## Cut Layers

'Cut' layers include only the outlines of specific objects (e.g., for use in laser or mechanical cutting). Using cut layers is easy: if any node on a layer has "$CUT" applied to it, a cut layer will be generated for that layer. Only nodes with "$CUT" will have their outlines included in the cut layer. Additionally, if "$CUT_OVERLAP" is called anywhere in the script, outlines will be allowed to overlap each other (an example follows below). As an important note, depending on what hardware is being used to perform cutting, cut layers generated as PDFs by this program may need to be vectorized into various other formats (such as .dxf). There are numerous freely available programs that can accomplish this online (such as pstoedit). In the circumstance that the developers of the cutting software failed to provide a means to import other than via specific commercial design software, the output PDF must be imported into that software, rasterized and vectorized there, and then imported into the cutting software. For instance, some programs will only function with Adobe Illustrator, and thus the PDF must be loaded, selected, rasterized, and then vectorized by using the Live Trace feature. Unfortunately, in cases where the cutting

hardware is hardcoded to only work with specific software, there may be no freely available workaround. Thus, we suggest making cutting hardware purchasing decisions with caution. Extensive information and tutorials about these pathways are available online, and the exact procedure will vary depending on which cutting robot is used.

| **$CUT Commands** | |
|---|---|
| **$CUT** | If applied to a node, a cut layer will be generated for that node's layer, and that node will have its outline included. |
| **$CUT_OVERLAP** | If called anytime before $FILL, the cut layer will not eliminate overlapping edges. |

Below is an example of $CUT commands in use:

```
>0
    $CIRCLE 3
    $BUFFER 2
    $CUT
    >LEFT
        $SPACE 6
        $CIRCLE 0.5
        $BUFFER 2
        $CUT
>0
    >RIGHT
        $RECT 12 2
        $BUFFER 2 2
        $CUT
>0
    $FILL 20 example11
    $CUT_OVERLAP
    $FILL 20 example11_overlap
```

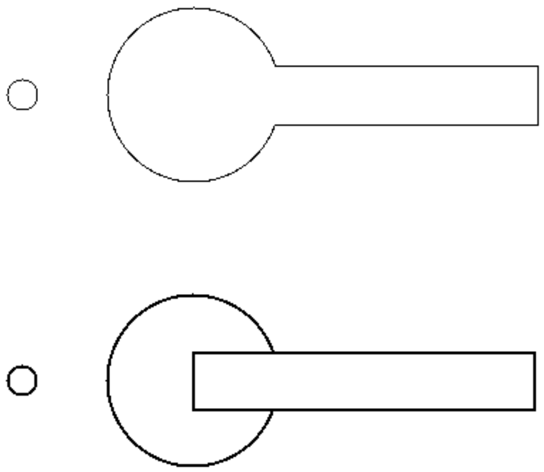

## Buffer Boxing

If snap-to-shape buffering is not desired, a buffer box system is also available. If $BUFFER_BOX is called anywhere in the script before $FILL, the entire background will be black instead of white. Additionally, $BUFFER_BOX can take a width and height of extra white space to put on each side of the image.

| **$BUFFER_BOX Commands** | |
|---|---|
| **$BUFFER_BOX**<br>**$BUFFER_BOX len # wid #** | Replaces the background with black. If a length and width are specified, will add that much white space on each side of the image. |

Below is an example of $BUFFER_BOX commands in use:

```
>0
        $CIRCLE 3
        $BUFFER 2
        >LEFT
                $SPACE 6
                $CIRCLE 0.5
                $BUFFER 2
>0
        >RIGHT
                $RECT 12 2
                $BUFFER 2 2
>0
        $FILL 20 example12
        $BUFFER_BOX
        $FILL 20 example12_box
        $BUFFER_BOX len 4 wid 4
        $FILL 20 example12_box_space
```

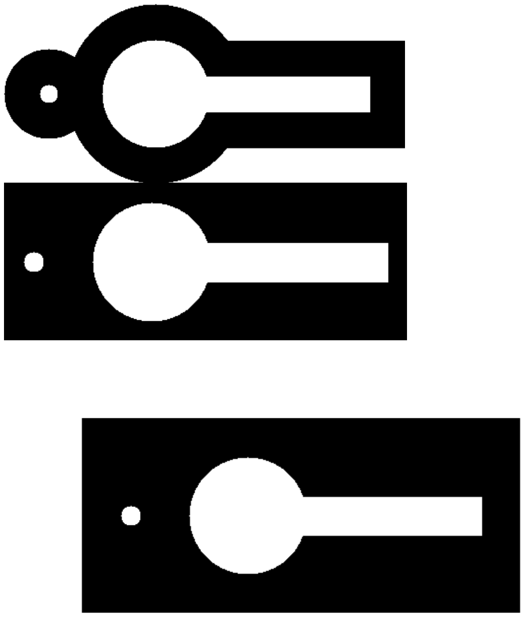

## Polygons

All simple polygons—closed-form objects whose lines do not intersect—are possible within the system; however, up until this point the syntax for creating these structures has not been discussed. Polygons are somewhat more complicated to create than circles or rectangles, as necessitated by their flexibility. To create a polygon, first a node must be marked via the $POLY command and then each vertex must be specified in arbitrary space. The arbitrary space of each polygon has its center at (0,0), and the positive X direction is in the direction of the node's angle.

| **Polygon Commands** | |
|---|---|
| **$POLY**<br>**$POLYGON** | Marks the node as a polygon. |
| **$VERTEX x # y #**<br>**$VERTEX x # y # type #**<br>**$VERTEX x # y # type # buffer #**<br>**$VERTEX x # y # type # buffer # extra #**<br>**$VERTEX** can be substituted with **$VEX**, **$VERT** or **$VX** | Adds a vertex to this node's polygon. Vertices must be added in succession (i.e., it is assumed that the new vertex is connected to the last vertex added). Each vertex must have an x and y position, and optionally can have a connection type, buffer size and extra length. |
| **Vertex Types** | The vertex type refers to its connection with the next vertex. This connection can be a straight or curved line, and is straight by default.<br>0 : straight<br>1 : curved, keeping higher<br>2 : curved, keeping lower |
| **$ROT_CENTER x # y #** | Moves the rotation center of the polygon from its arbitrary space origin of (0,0) to a new point. |
| **$OUTLINE** | Marks the polygon to not be filled in, and only be an outline. |
| **$BUFFER radius #** | When called, makes all the points of the polygon have the same buffer radius. |
| **$EXTRA radius #** | When called, makes all the points of the polygon have the same extra radius. |

Below are some examples of how to construct polygons:

| | |
|---|---|
| $POLY<br>$VERTEX 0 0<br>$VERTEX 0 3<br>$VERTEX 3 0 | 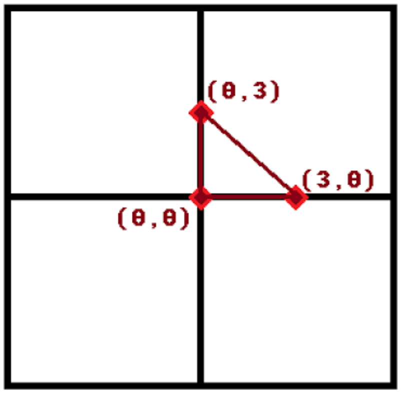<br> |
| $POLY<br>$VERTEX 0 2<br>$VERTEX 8 2<br>$VERTEX 8 -2<br>$VERTEX 0 -2 | 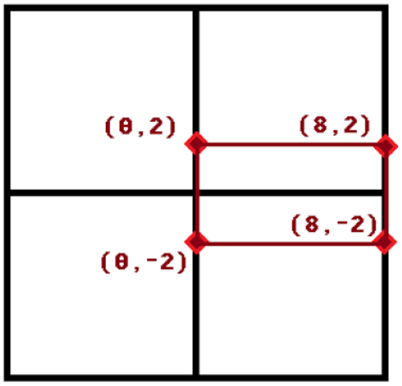<br> |
| $POLY<br>$VERTEX 0 3 concave_down 1<br>$VERTEX 3 0 concave_up 2<br>$VERTEX 0 -3 concave_up 2<br>$VERTEX -3 0 concave_down 1 | 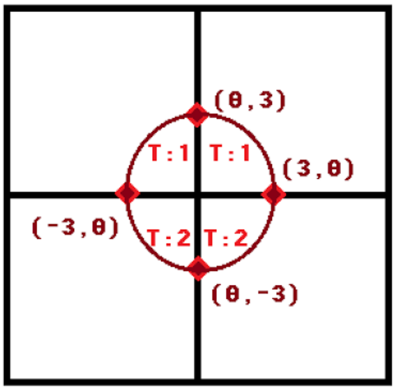<br> |

In short, each polygon is centered on the origin and extends forward in the positive X direction. The coordinates of each polygon are then mapped over the node's position and are then rotated by the node's angle. For example, if the node position is (10,5) then the polygon's (0,3) point becomes (10,8). It is often useful to draw out polygons first on graph paper, and then translate the points to code. References will also be discussed later as a technique for making reusable polygon templates, which can save a significant amount of time in this process.

In practice, creating polygons works in the same fashion. Note that all of the following examples are on node 0 and thus are fixed to an angle of 0 degrees.

| | |
|---|---|
| >0<br>$POLY<br>$VERTEX 0 0<br>$VERTEX 8 0<br>$VERTEX 0 4<br>$BUFFER 1<br>>0<br>$FILL 20 example13 | >0<br>$POLY<br>$VERTEX 0 0<br>$VERTEX 8 0<br>$VERTEX 8 4<br>$VERTEX 0 4<br>$BUFFER 1<br>>0<br>$FILL 20 example 14 |
| 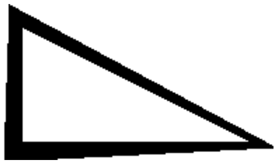 | 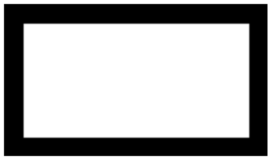 |
| >0<br>$POLY<br>$VERTEX 0 0<br>$VERTEX 4 -3<br>$VERTEX 8 0<br>$VERTEX 8 4<br>$VERTEX 0 4<br>$BUFFER 1<br>>0<br>$FILL 20 example15 | >0<br>$POLY<br>$VERTEX 0 0<br>$VERTEX 4 -3<br>$VERTEX 8 0<br>$VERTEX 8 4<br>$VERTEX 4 7<br>$VERTEX 0 4<br>$BUFFER 1<br>>0<br>$FILL 20 example16 |
| 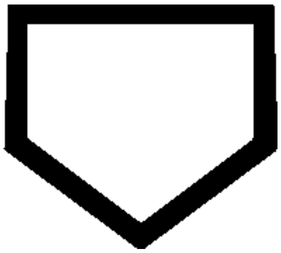 | 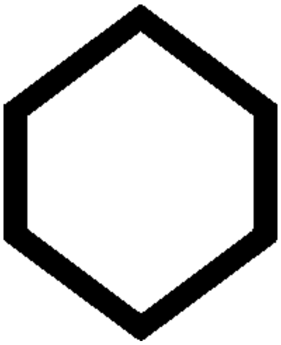 |

| | |
|---|---|
| >0<br>$POLY<br>$VERTEX 0 0<br>$VERTEX 3 2<br>$VERTEX (13 ^ 0.5) 0<br>$VERTEX 3 -2<br>$BUFFER 1<br>>0<br>$FILL 30 example17 | >0<br>$POLY<br>$VERTEX 0 0<br>$VERTEX 3 2 curvedown 1<br>$VERTEX (13 ^ 0.5) 0 curveup 2<br>$VERTEX 3 -2<br>$BUFFER 1<br>>0<br>$FILL 30 example18 |
| 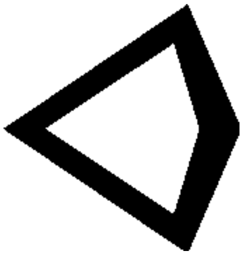 | 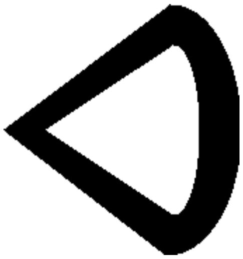 |
| >0<br>$POLY<br>$VERTEX 0 3 curvedown 1<br>$VERTEX 3 0 curveup 2<br>$VERTEX 0 -3 curveup 2<br>$VERTEX -3 0 curvedown 1<br>$BUFFER 1<br>>0<br>$FILL 30 example19 | >0<br>$POLY<br>>0<br>$POLY<br>$VERTEX 0 3 curvedown 1<br>$CURVE_WEIGHT 0.6<br><br>$VERTEX 3 0 curveup 2<br>$CURVE_WEIGHT 0.5<br><br>$VERTEX 0 -3 curveup 2<br>$CURVE_WEIGHT 0.4<br><br>$VERTEX -3 0 curvedown 1<br>$CURVE_WEIGHT 0.3<br><br>$BUFFER 1<br>>0<br>$FILL 30 example20 |
| 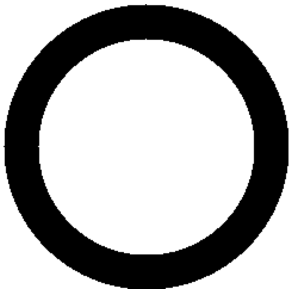 | 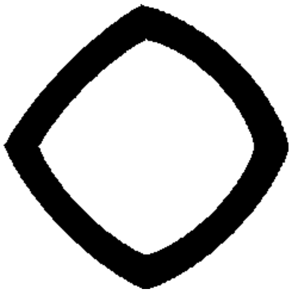 |

Of course, polygons can also be rotated if they are not on node 0:

```
>0
        >ANGLE 0
                $POLY
                $VERTEX 0 0
                $VERTEX 4 2
                $VERTEX 3 0
                $VERTEX 6 -2
                $BUFFER 1
>0
        >ANGLE 120
                $POLY
                $VERTEX 0 0
                $VERTEX 4 2
                $VERTEX 3 0
                $VERTEX 6 -2
                $BUFFER 1
>0
        >ANGLE 240
                $POLY
                $VERTEX 0 0
                $VERTEX 4 2
                $VERTEX 3 0
                $VERTEX 6 -2
                $BUFFER 1
>0
        $FILL 30 example21
```

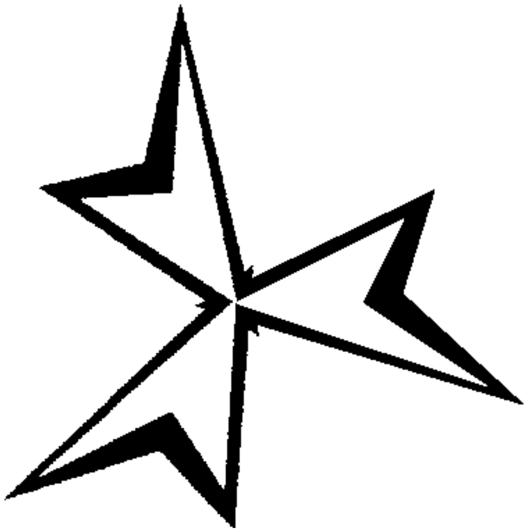

## Temp and Back

Additionally, there are two advanced '>' commands:

| **>TEMP and >BACK Commands** | |
|---|---|
| **$TEMP #** | Marks this node with a temporary place. |
| **>TEMP #** | Returns to a node marked with a given temporary ID. |
| **>BACK #** | Goes backwards in the list of previous nodes, a given number of spaces. For example, ">BACK 1" goes to the node before this one. |

>TEMP functions exactly the same as >PLACE, except the list of temporary nodes is meant to be overwritten and reused, whereas the list of placed nodes should be constant and final. For instance:

```
>0
	$CIRCLE 2
	$BUFFER 1
	>LEFT
		$RECT 8 2
		$BUFFER 1 1
		$TEMP 0
		>LEFT
			$RECT 8 2
			$BUFFER 1 1
	>TEMP 0
		>UP
			$RECT 8 2
			$BUFFER 1 1
>0
	$FILL 20 example22
```

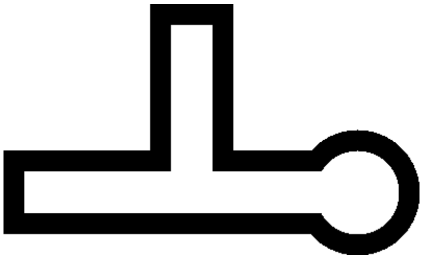

>BACK functions uniquely. As nodes are added, a list of the last 64 nodes is kept by the Interpreter. >BACK accesses this list and recalls a node from it. For instance:

```
>0
	$CIRCLE 2
	$BUFFER 1
	>LEFT
		$RECT 8 2
		$BUFFER 1 1
		$TEMP 0
		>LEFT
			$RECT 8 2
			$BUFFER 1 1
	>BACK 1
		>UP
			$RECT 8 2
			$BUFFER 1 1
>0
	$FILL 20 example23
```

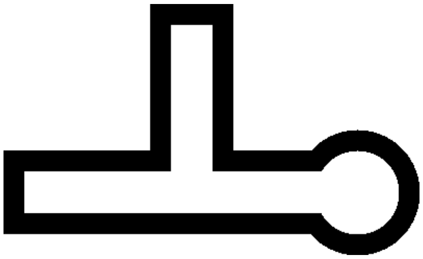

These commands are mostly used in the construction of references, which require non-fixed and relative ways of accessing nodes.

## Operations & Variables

Operations allow numbers to be modified and recalled, while variables allow numbers to be stored. For instance:

```
>0
	$VAR 0 2
	$CIRCLE (VAR 0)
	$BUFFER 1
	>LEFT
		$RECT ((VAR 0) * 3) (VAR 0)
		$BUFFER 1 1
		$PRECISE
		>LEFT
			$CIRCLE ((VAR 0) + 1)
			$BUFFER 1
>0
	$FILL 30 example24
```

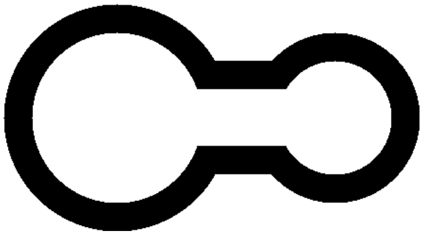

The advantage of using variables is, if measurements are based around variables, changing just one variable can change the whole design appropriately. The above design, varying variable 0, looks as such:

| Var 0 = 4 | Var 0 = 0.5 |
|---|---|
| 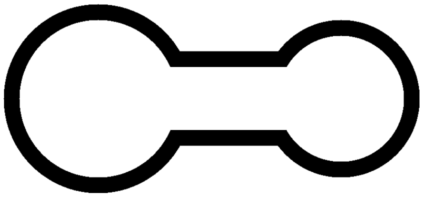 | 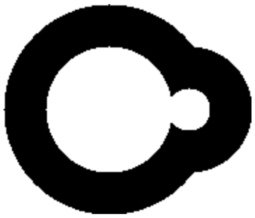 |

Though a full list of operations is included in the Glossary, a few basic operations follow:

| Operations and Variables Commands | |
|---|---|
| **$VAR # #** | Sets a variable to a value. The first number is the variable ID, the second number is the value (e.g. “$VAR 0 4” sets variable 0 to equal the number 4). **As an important note, it is strongly suggested to avoid using variables 0-9, as they are often used by reference scripts.** |
| **(# + #)**<br>**(# - #)**<br>**(# * #)**<br>**(# / #)** | Addition, subtraction, multiplication, division |
| **(# ^ #)** | Exponent |
| **(VAR #)** | Returns value of a variable with the given ID. |

Operations must always follow the syntax “(# ? #)” or “(? #)”. Spaces are necessary between operation inputs. Operations can be nested (e.g. “(2 + (3 - 1))”). Properties from nodes can also be accessed:

| Node Operations | |
|---|---|
| **(# . ?)** | Returns a property of a node, where # is a node ID (usually given by the place ID, (BACK #), or (TEMP #)) and ? is a property ID (such as “COLOR_RED”, “RECT_LENGTH”, or “ANGLE”).<br>See the Glossary for a full list of property IDs. |
| **Place ID** | If a place ID is used as the number in the above operation, the node at that place will be used. |
| **(TEMP #)** | Returns the node at the temporary ID. |
| **(BACK #)** | Returns the node that is the given number of spaces back in the previous node list. |
| **(# to #)** | Gives the distance between two nodes. The numbers in this operation are two node IDs, either place IDs or given by (TEMP #) or (BACK #). |

For instance:

```
>0
        $CIRCLE 4
        $BUFFER 1 1
        >LEFT
                $PRECISE
                $RECT ((BACK 1) . RADIUS)
2
                $BUFFER 1 1
>0
        $FILL 30 example25
```

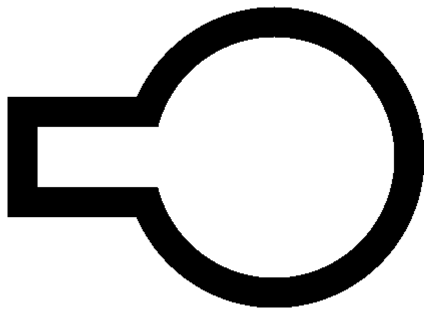

In this case, the rectangle will always be as long as the radius of the circle, since it references that value for its length. This is seen if that radius is varied, as follows:

| $CIRCLE 1 | $CIRCLE 8 |
| --- | --- |
| 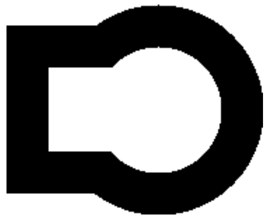 | 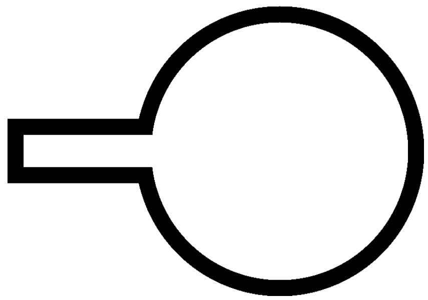 |

## References

Referencing allows for blocks of code to be repeated. To do so, a reference of code is constructed and then called upon (i.e., "referenced") to be repeated later on in a script. A reference is constructed as follows:

```
{ title
        "code goes here
}
```

And referenced as "{ title }". For instance:

```
{ circle
        $CIRCLE 2
        $BUFFER 1
        $COLOR 255 0 0
}
>0
        { circle }
        $FILL 20 example26
```

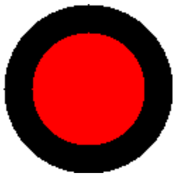

Of course, the above example doesn't show the power of referencing; consider the following, instead:

```
{ rectref
        >UP
                $RECT 4 2
                $BUFFER 1 1
                >LEFT
                        $RECT 6 2
                        $BUFFER 1 1
}
>0
        { rectref } x 4
        $FILL 20 example27
```

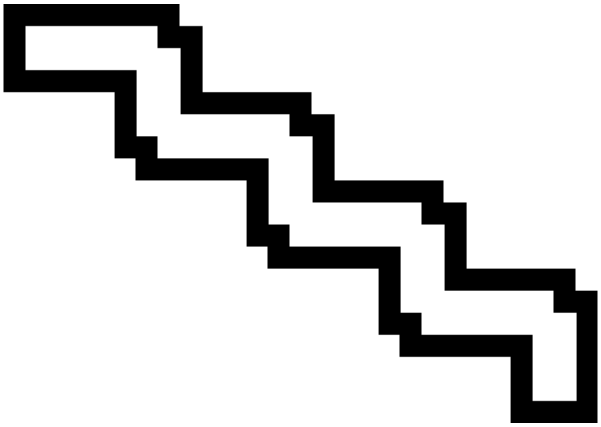

This example shows a key feature of referencing: simple repetition. The image above has 8 shapes in it, but only 2 have been described in the script. The "x 4" at the end of the reference callback is part of the reference syntax, and specifies that the reference will be repeated 4 times. The full reference specification follows:

| **Reference Commands** | |
|---|---|
| **{ title**<br>“code here<br>**}** | Specifies a reference. Should be placed at the top of the script, as it cannot be used before it is defined. |
| **{ title }**<br>**{ title }** **x #**<br>**{ title } ANGLE #**<br>**{ title } VARS # # # # # # # # #**<br>**{ title } x # VARS # # # # # # # # #**<br>**{ title } ANGLE # VARS # # # # # # # # #**<br>**{ title } x # ANGLE # VARS # # # # # # # # #** | Calls back a reference for usage.<br>A reference can be repeated a number of times by appending “x #” to the reference callback.<br>A reference can have its angles adjusted by an amount by appending “ANGLE #” (e.g. “ANGLE 30” would add 30 degrees to each angle in the reference).<br>A reference can also set variables 0 through 8 from its callback by appending “VARS” and up to 9 numbers (e.g. “VARS 4 9” would set variable 0 to 4 and variable 1 to 9). |

By appending “ANGLE #”, a reference can have its angle adjusted. For instance:

```
{ rectref
        >RIGHT
                $RECT 8 2
                $BUFFER 1 1
                >RIGHT
                        $CIRCLE 1
                        $BUFFER 1.1
}
>0
        { rectref } ANGLE 0
        { rectref } ANGLE 60
        { rectref } ANGLE 120
        { rectref } ANGLE 180
        { rectref } ANGLE 240
        { rectref } ANGLE 300
        $FILL 20 example28
```

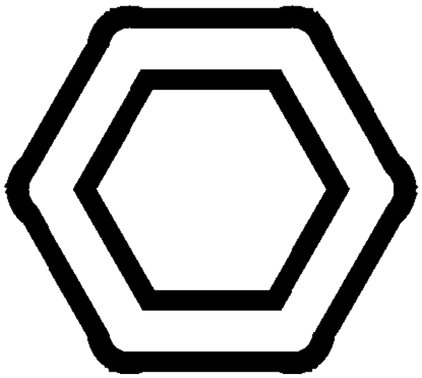

In this case, the same reference is repeated at differing angles to make a loop. By using variables, we can condense this further:

```
{ rectref
        >ANGLE (VAR 0)
                $RECT 8 2
                $BUFFER 1 1
                >ANGLE (VAR 0)
                        $CIRCLE 1
                        $BUFFER 1.1
“set var0 = itself + 60
                        $VAR 0 ((VAR 0) +
60)
}
>0
        $VAR 0 0
        { rectref } x 6
        $FILL 20 example29
```

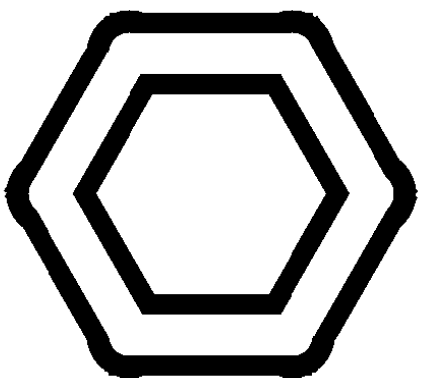

And, with this setup, it is trivial to make a wider loop:

```
{ rectref
        >ANGLE (VAR 0)
                $RECT 4 2
                $BUFFER 1 1
                >ANGLE (VAR 0)
                        $CIRCLE 1
                        $BUFFER 1.1
                        $VAR 0 ((VAR 0) +
20)
}
>0
        $VAR 0 0
        { rectref } x 18
        $FILL 20 example30
```

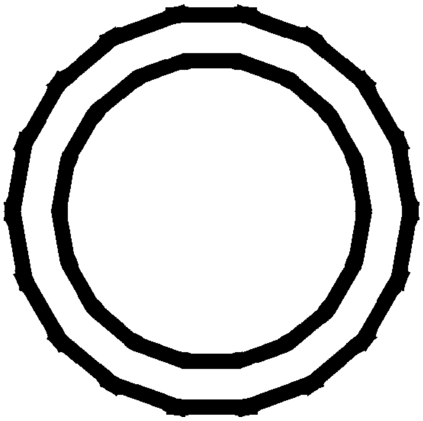

Note as well that it is possible to 'nest' references within one another:

```
{ rectref
	>ANGLE (VAR 0)
		$RECT 4 2
		$BUFFER 1 1
		{ nestedref }
		>ANGLE (VAR 0)
			$CIRCLE 1
			$BUFFER 1.1
			$VAR 0 ((VAR 0) +
20)
}
{ nestedref
	>ANGLE ((VAR 0) - 90)
		$RECT 5 1.5
		$BUFFER 1 1
		>BACK 1
		" Go back to where this ref
		" started.
}
>0
	$VAR 0 0
	{ rectref } x 18
	$FILL 20 example31
```

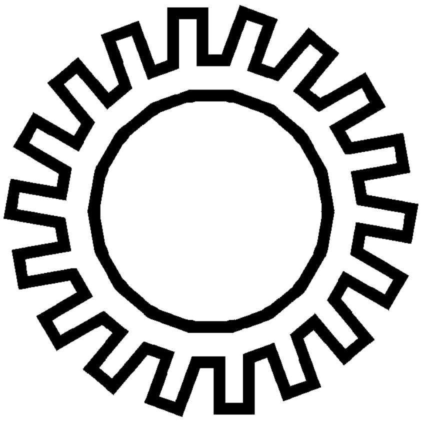

Note also that in the above example the nested reference appears to be called in the rectangle reference before it is defined; in actuality, the rectangle reference itself isn't called until after the nested reference is defined, so the calls within that reference are not processed until that point. Variables are used to pass parameters into references. Most commonly, this allows for shape-related references to be scaled up or down in size. For instance:

```
{ tri_eq
        $POLY
                $VAR 1 ((((VAR 0) ^ 2) - (((VAR 0) / 2) ^ 2)) ^ 0.5)
                " automatically determines the height of the third vertex
                " & stores that value in VAR 1
                $VERTEX 0 ((VAR 0) / 2)
                $VERTEX (VAR 1) 0
                $VERTEX 0 ((VAR 0) / -2)
}
>0
        >UP
                $SPACE 2
                { tri_eq } sidelen 3
                $BUFFER 1
>0
        >LEFT
                $SPACE 2
                { tri_eq } sidelen 1
                $BUFFER 1
>0
        >RIGHT
                $SPACE 2
                { tri_eq } sidelen 2
                $BUFFER 1
>0
        >DOWN
                $SPACE 2
                { tri_eq } sidelen 0.5
                $BUFFER 0.5
>0
        $FILL 30 example32
```

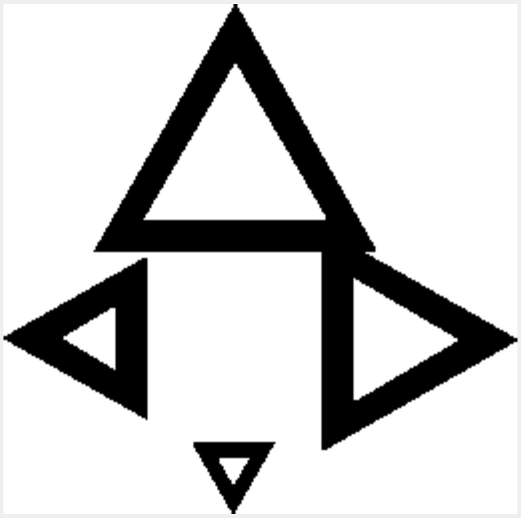

References in this fashion save the user a lot of work, as this equilateral triangle script automatically performs all of the trigonometric calculations involved with finding the height of the triangle, for instance. When building complex shapes, it is often wiser to make reference scripts that will handle the calculations than to create each shape by hand. Additionally, references can be shared and re-used by other users. The program itself comes with several scripts, such as the one above, for making basic shapes. As a final precaution, it should be noted that nesting only functions up to the 16th layer of nesting.

## Combined-Layers

Combined-Layers are used to make arrays of layers; that is, combining multiple layers into a single image. This approach finds applications in designing devices that are assembled by folding a single piece of patterned paper (i.e., origami). As per usual, it is simpler to visualize this concept. Consider the following script, which produces two regular layers and the subsequent script that combines them:

```
>0
        $CIRCLE 2
        $BUFFER 1
        >RIGHT
                $RECT 6 2
                $BUFFER 1 1
                >RIGHT
                        $CIRCLE 2.5
                        $BUFFER 1
                        $PLACE 1
>0
        >LAYER 1
                $CIRCLE 2
                $BUFFER 1
>1
        >LAYER 1
                $CIRCLE 2.5
                $BUFFER 1
>0
        $FILL 30 example33
```

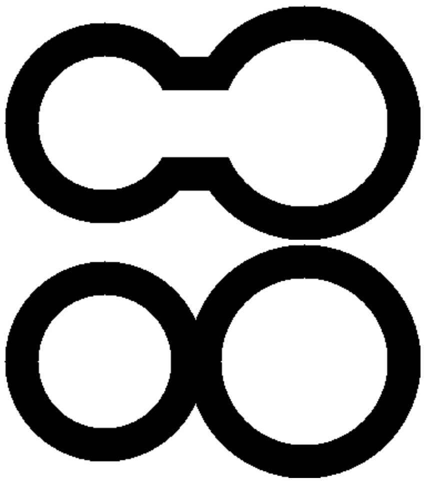

```
#COMBINE 0
        #ID 0
                #LAYER 0
        #ID 1
                #LAYER 1
                #LEFT 0
        #ID 2
                #LAYER 0
                #DOWN 1
                #ROTATE 90
        #ID 3
                #LAYER 1
                #LEFT 2
                #ROTATE 90
        #SQUARESPACE
        #BOX
        #FILL combined33
```

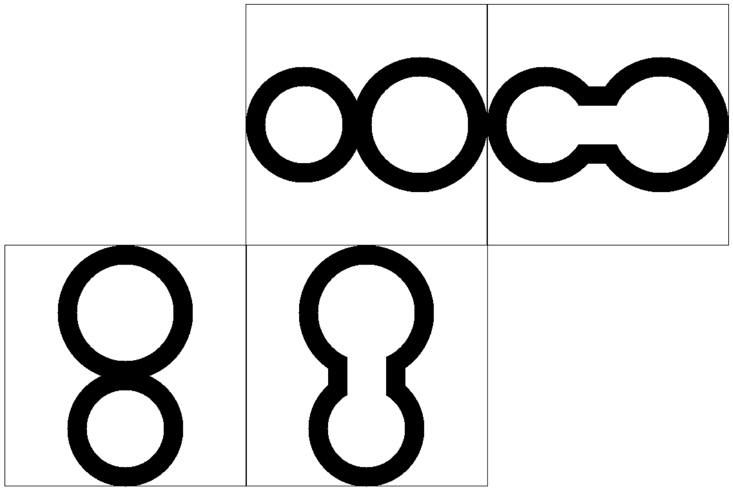

```
#COMBINE 1
	#ID 0
		#LAYER 0
	#ID 1
		#LAYER 1
		#LEFT 0
	#ID 2
		#LAYER 0
		#DOWN 1
		#ROTATE 90
	#ID 3
		#LAYER 1
		#LEFT 2
		#ROTATE 90
	#SQUARESPACE
	#CENTER 0
	#BOX
	#FILL combined33B
```

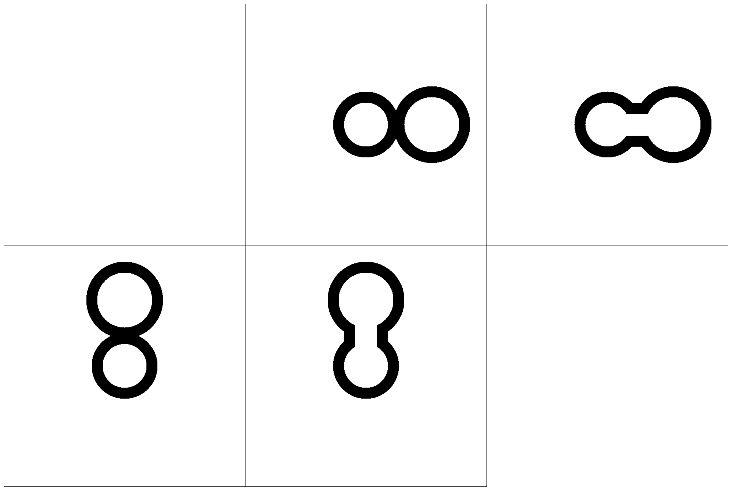

Combined-layers follow the same syntax rules as the rest of the scripting language, though they do have a unique structure. First, each combined layer (each script can support up to 64 combined layers) must be initialized with a “#COMBINE #” command. Combines should start at 0 and increase (e.g. the second combined layer is “#COMBINE 1”). Next, the combine is filled with “#ID #” commands, each of which represents a layer. ID’s are placed relative to each other via the “#LEFT #”, “#UP #”, “#RIGHT #”, and “#DOWN #” commands, where the # is the ID that this new ID is attached to. For instance, in the above example “#ID 1” has “#LEFT 0” which means it is placed to the left of “#ID 0”. Finally, the combine’s properties are set and it is “#FILL”ed. “#SQUARESPACE” makes each cell in the combined-layer a square, and should be used most of the time. “#BOX” draws outlines around each cell in the array. Using the “#CENTER #” function as in the third case ensures that a specified node (i.e., the node number used in the function) will be at the center of each cell, regardless of how the layer has been rotated. Centering is often necessary if the combined layer features rotation, as otherwise the alignment can become undone.

| Combined-Layer Commands | |
|---|---|
| **#COMBINE #** | Defines a new combined-layer with the given ID. |
| **#ID #** | Adds a layer to the combined-layer array. |
| **#LAYER #** | Defines which layer the most recent ID references. |
| **#LEFT #**<br>**#UP #**<br>**#RIGHT #**<br>**#DOWN #** | Determines where the most recent ID is placed. The # is the ID that this ID is attached to (e.g. "#UP 2" means this ID is placed up above ID 2). |
| **#ROTATE #** | Rotates the most recent ID. Only accepts 90, 180 or 270. |
| **#FLIPX**<br>**#FLIPY** | Flips the most recent ID horizontally (X) or vertically (Y). |
| **#TEMP #** | As before, used to make temporary markers. The only use of this command in the context of combined-layers is to reference the properties of other IDs via the (TEMP #) command. |
| **#VAR # #** | As before, used to set variables. |
| **#BOX** | Draws an outline around each cell in the array. |
| **#SQUARESPACE** | Forces each cell to be square-sized. |
| **#FILL title** | Creates the combined-layer image with the given title. |
| **#CENTER #** | Centers the layers over a certain point, specifically the ID of a node. This means that flipped or rotated layers in the array will be aligned such that, were they to be overlaid with the un-rotated layer, this center point would be in the center of each (e.g. if they were folded onto each other, those points would align). |

As in the first example, combined-layer definitions are always attached at the bottom of a script, *after* the $FILL command. Without using "#SQUARESPACE", each new cell is aligned to the center of its source. For instance:

```
>0
	$CIRCLE 2
	$BUFFER 1
	>RIGHT
		$RECT 12 2
		$BUFFER 1 1
>0
	$FILL 30 example34
```

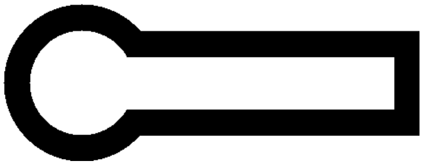

```
#COMBINE 0
	#ID 0
		#LAYER 0
		#ROTATE 90
	#ID 1
		#LAYER 0
		#LEFT 0
	#ID 2
		#LAYER 0
		#DOWN 1
		#ROTATE 90
	#ID 3
		#LAYER 0
		#DOWN 2
	#BOX
	#FILL combined34
```

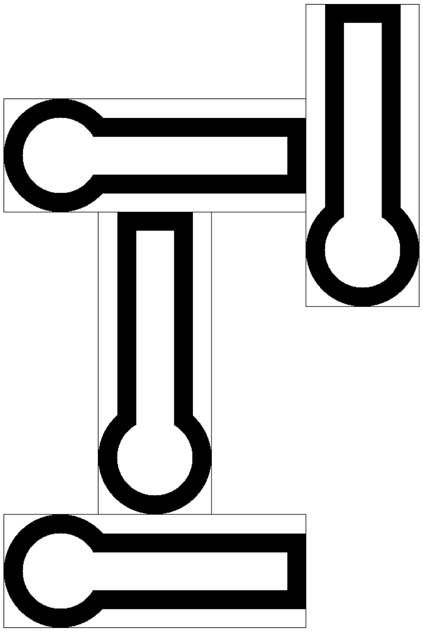

Due to this, it can be difficult to align layers without using squares, and thus the utility of "#SQUARESPACE" should be apparent. Additionally, rotated layers are not guaranteed to align, so it is suggested to create each layer individually rather than rotating, and the same considerations should be made for flipping. References can be used for combined-layers in the same way that they would be used for other parts of the script, however the "(COMBINE #)" operator must be introduced: the "(TEMP #)" and "(BACK #)" operators work for combined-layers, but the values they return must be parsed by "(COMBINE #)" in order to give the actual ID value. One exception, however: to use the "(# . ?)" command, these values should not be parsed.

This being said, the "#ID" and "#UP", "#LEFT", "#RIGHT", "#DOWN" commands will automatically perform this conversion if necessary, so using "(COMBINE #)" is unnecessary in those cases. For instance:

```
>0
        $CIRCLE 2
        $BUFFER 1
        >RIGHT
                $RECT 12 2
                $BUFFER 1 1
>0
        $FILL 30 example35
```

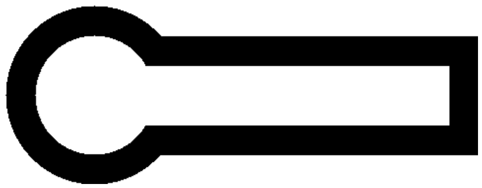

```
{ combstack
        #ID ((BACK 0) + 1)
                #LAYER 0
                #DOWN (BACK 1)
}
#COMBINE 0
        #ID 0
                #LAYER 0
        { combstack } x 4
        #ID ((BACK 0) + 1)
                #LAYER 0
                #RIGHT 0
        { combstack } x 4
        #BOX
        #FILL 35combined0
```

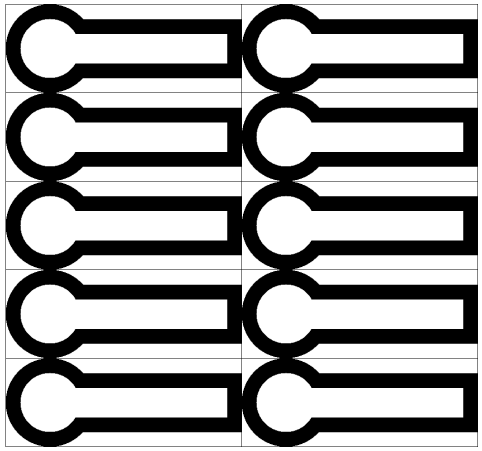

It is further worth noting that, as before, these references may be nested:

```
>0
        $CIRCLE 2
        $BUFFER 1
        >RIGHT
                $RECT 12 2
                $BUFFER 1 1
>0
        $FILL 30 example36
```

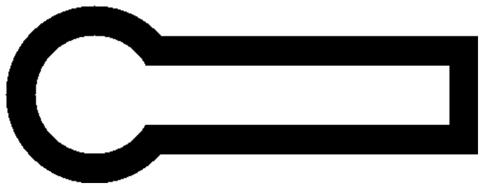

```
{ combstack
        #ID ((BACK 0) + 1)
                #LAYER 0
                #DOWN (TEMP 1)
                #TEMP 1
                { combstack2 } x 4
}
{ combstack2
        #ID ((BACK 0) + 1)
                #LAYER 0
                #RIGHT (BACK 1)
}
#COMBINE 0
        " must set up initial node
        #ID 0
                #LAYER 0
                #TEMP 1
        " make first row
        { combstack2 } x 4
        " make the rest:
        { combstack } x 8
        #BOX
        #FILL 36combined0
```

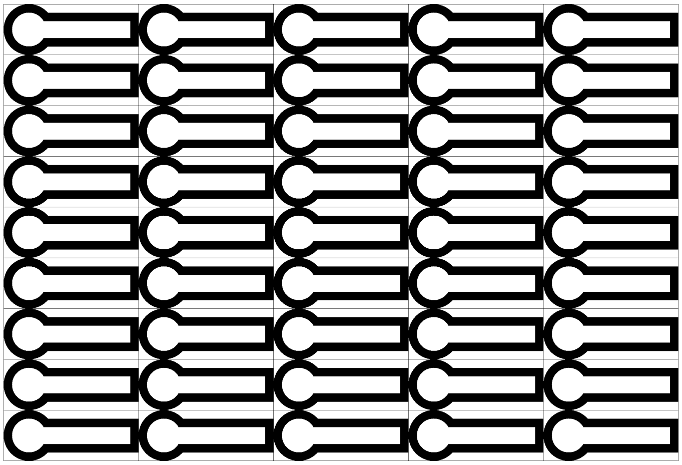

It is absolutely critical that loops where each ID is its own ancestor are avoided, as these won't compile. Also, note the use of "#TEMP" above to mark a node to be a source for later.

## Printing and PDFs

For each image generated by the Interpreter, a PDF is automatically created. By default, these PDFs are of the standard U.S. paper size (8.5 in. × 11 in.) with half-inch margins. The Interpreter will automatically place as many devices as it can per sheet by tiling. These PDFs can be then be printed and assembled into devices. These parameters—the page size and margins—can be tweaked in the device script:

| **PDF Commands** | |
|---|---|
| **$PDF_OFF** | Disables PDF generation for this device. |
| **$PDF_SIZE length # width #** | Sets the PDF page size in pixels. |
| **$PDF_SIZE_IN len # wid #** | Sets the PDF page size in inches. |
| **$PDF_SIZE_CM len # wid #** | Sets the PDF page size in centimeters. |
| **$PDF_MARGIN len # wid #** | Sets the horizontal and vertical page margins in pixels. |
| **$PDF_MARGIN_IN len # wid #** | Sets the page margins in inches. |
| **$PDF_MARGIN_CM len # wid #** | Sets the page margins in centimeters. |

When using PDFs, note that AutoPAD exports images at a resolution of 300 pixels per inch. Using $FILL conversions of these sizes allows for the dimensions of the device to be in those respective units. For instance, using a $FILL of 300 means that each dimension of the device is in inches: a 2 × 4 rectangle is now a 2 × 4 inch rectangle, and so on. Similarly, a $FILL of 11.84 would make that 2 × 4 rectangle a 2 × 4 mm rectangle. In general, best practices are to pick a device scale first, before doing any other part of the design.
